# The effect of previously acquired languages on third language acquisition

**DOI:** 10.1016/j.heliyon.2024.e26202

**Published:** 2024-02-14

**Authors:** Xiaoyu Luan, Masakazu kuno, Ayaka Sugawara, Yayoi Kawasaki, Eriko Sugimori

**Affiliations:** a Graduate School of Human Sciences, Waseda University, 2-579-15 Mikajima, Tokorozawa-shi, Saitama-ken, Japan; b Faculty of Education and Integrated Arts and Sciences, Waseda University, 1-104 Totsukamachi, Shinjuku-ku, Tokyo, Japan; c Faculty of Science and Engineering, Waseda University, 3-4-1 Ookubo, Shinjuku-ku, Tokyo, Japan; d Faculty of Human Sciences, Waseda University, 2-579-15 Mikajima, Tokorozawa-shi, Saitama-ken, Japan

**Keywords:** Third language acquisition, Cognitive economy, Typological proximity, Sentence structure, Sloppy reading, Relative clauses

## Abstract

Our study explores how previously acquired languages affect third language (L3) acquisition. The learning and control groups composed adpositional phrases and relative clauses, and then judged sentences with strict/sloppy readings presented in their L3. The results showed that native Japanese learners of Chinese were more influenced by the second language (English) for adpositional phrases and relative clauses than were native Chinese learners of Japanese, although both were influenced more by their native than second language (English) in strict/sloppy interpretation. This indicates that L3 acquisition can be influenced by all previously acquired languages and that the interrelationship between the positions of subgrammars in a sentence structure may influence learners’ assessment of the structural similarity of the selected subgrammars, making it an important trigger for non-facilitative transfer. Overall, structural similarities played a stronger role than did typological proximity. This study differs from traditional models of L3 acquisition that focus on wholesale or property transfer by beginning with an investigation of the conditions under which non-facilitative transfers occur. These two perspectives are integrated in terms of cognitive economy, pointing to a more promising direction for L3 acquisition research in the future.

## 1 Introduction

### 1.1 The effect of previously acquired languages on third language acquisition

Third language acquisition (L3A) and second language acquisition (SLA) mechanisms differ [1]. While SLA is influenced only by the native language (L1), all previously acquired languages (PAL) can influence L3A [2]. Research on L3A is scarce compared to that on SLA, although globalization is increasing the demand to acquire L3 and even more languages (Ln). Therefore, a reliable L3A model is needed to guide L3 learning and teaching, the demand for which is increasing. Current L3A models aim to explain the factors that play the most significant role in linguistic transfer and, thus, elucidate the L3A mechanisms. Additionally, most of them discuss the source, form, and impact of transfer on L3A. Table 1 summarizes the characteristics of the current mainstream L3A models from the abovementioned four perspectives.

**Table 1:** Summary of the current L3A models.

	Variable with Ultimately Explanatory Status	the Source of Transfer	the Form of Transfer	the Impact of Transfer
The L1 privileged role theory,	L1	L1 only	Not clearly defined, but overall, a wholesale transfer	Positive and non-facilitative transfer
The L2 status factor hypothesis	L2	L2 only	Not clearly defined, but overall, a wholesale transfer	Not clearly defined, but overall, both positive and non-facilitative transfers are possible
The Typological Primacy Model	(Psychological) Typological proximity	L1 or L2	Wholesale transfer	Positive and non-facilitative transfer
The Cumulative Enhancement Model	Not clearly defined, but overall depends on typological differences	L1 & L2	Property-by-property transfer	Only positive transfer
Hybrid transfer models (Models advocating property-by-property transition)	Structural similarity	L1 & L2	Property-by-property transfer	Positive and non-facilitative transfer

Note: Hybrid transfer models contain the Language Proximity Model and the Scalpel Model.

These L3A models can be categorized into two main groups. The first group claims that the order of acquisition is the most dominant influence on transfer and consists of the L1 privileged role theory and the L2 status factor hypothesis (L2SF). Among them, the key concept of the L1 privileged role theory is the dominance of the native language in all subsequent language acquisition [3,4], with the mechanism that the native language, as the strongest and also the most active language, should therefore be the most determining factor in learning any other non-native language. Several studies have supported the claim [5,6]. In contrast to the L1 privileged role theory, the L2SF proposes that L2 is the dominant factor in L3A [7]. The mechanism inherent in this theory is that L1 grammar is considered to be essentially procedural memory, whereas L2 and L3 acquisition in adulthood is essentially declarative memory [8], and therefore learners will use a similar learning approach to L2 when learning L3. Falk and Bardel's [2] work in the field of morphosyntax, as well as the work of Ghezlou, Koosha and Lotfi's [9] experimental results, support this theory.

Unlike the models mentioned above, models advocating typological proximity/structural similarity all assume that transfer can come from either L1 or L2, and among such models, the Typological Primacy Model (TPM) advocates a wholesale transfer based on the view of cognitive economy [[10], [11], [12]]. Typological proximity in TPM mainly refers to psychological typology, i.e., the learner's subjective perception of typological similarity between languages [11]. Rothman [12] suggests that when inputting L3, learners may unconsciously judge the similarity between the target language and each of the pre-acquired languages in the following order:

Lexicon → Phonetics/phonology → Morphology → Syntactic

When the similarity between one of the previously acquired languages and L3 reaches the threshold, the language is determined to be similar to L3, and its features will be fully transferred to L3. Thus, not only facilitative but also non-facilitative transfers can be observed in L3A. Conversely, unlike the TPM, the other three models that focus on typological proximity/structural similarity—the Cumulative Enhancement Model (CEM) [13], the Language Proximity Model (LPM) [14], and the Scalpel Model (SM) [15]—all argue against wholesale transfer. Among them, the CEM, although it does not explicitly discuss the way of transfer (overall or property), rejects non-facilitative transfer from PALs because of its principle of maintaining non-redundancy and maximum facilitation during acquisition, and in this theoretical framework, a wholesale transfer is unacceptable. Several studies have been conducted to support this model [16,17]. Unlike the CEM, the other two models, LPM and SM, have similar assumptions in the sense that they both explicitly claim that both L1 and L2 transfers can occur progressively in terms of properties in L3A and that this transfer is selective. They also assume that typological and abstract structural similarities between languages are important factors influencing the transfer from PAL to L3A. Among the hybrid transfer models, the basic principle of LPM is that each language contains several identifiable linguistic properties. Learners can create rule sets for each subgrammar, which are compatible with elements of all learned languages [18]. Cross-linguistic influence depends on the proximity of particular structures between languages. When a learner comprehends or produces a rule set in L3, the corresponding structural representations of both L1 and L2 are also activated and compete with each other for linguistic proximity. The learner selects the PAL structure with the higher degree of activation and transfers it to L3. Another model, SM, incorporates the properties of the LPM and simply builds on its theoretical foundation by stating that in addition to structural proximity, additional factors such as the amount of use of each language, construction frequency, and processing complexity are also likely to have an impact on the occurrence of transfer and that attribute transfers can be carried out with “scalpel-like precision” [15]. Considering that in terms of content, SM is more of a complementary illustration based on the LPM theory, and the two are not fundamentally different from each other in terms of the four main perspectives shown in Tables 1 and in this study, instead of deliberately distinguishing between the two models, they are collectively referred to as the hybrid transfer models. Several studies have presented relevant evidence supporting these models [[19], [20], [21]].

Studies supporting L1 privileged role theory [5,6] and L2SF [9,22,23] counter each other, while confirmed non-facilitative transfer from PAL opposes CEM [24,25]. The remaining TPM and hybrid transfer models are currently at the heart of the L3A model debate [26,27]. Therefore, the first aim of this study is to investigate TPM and hybrid transfer models to determine whether transfers from PAL are overall or property-by-property. However, due to the limited number of subgrammars that can be examined in a single experiment, even if only transfer from either L1 or L2 is observed in the experiment, it is not certain that other unexamined subgrammars are not influenced by another PAL. Therefore, the most efficient way to design experiments is to conduct a design with the potential to observe both PALs simultaneously, although this is not meant to presuppose a position that supports hybrid transfer models.

Note that a primary purpose of studying the effects of PAL on target language acquisition is to minimize the occurrence of non-facilitative transfers, since such transfers will greatly affect the efficiency of acquisition. Therefore, a well-developed L3A model should be able to provide an explanation for the mechanism of the non-facilitative transfer occurrence, and only in this way can it bring positive guidance to the teaching practice in the classroom beyond the theoretical research. From this perspective, TPMs supporting wholesale transfer provide an explanation for the occurrence of non-facilitative transfer, i.e., because it is impossible for two identical languages to exist, the wholesale transfer of features from one language to another will obviously lead to the occurrence of non-facilitative transfer. Conversely, the hybrid transfer models are limited because they do not offer robust explanations for the mechanisms that cause non-facilitative transfer from PAL. When comparing the similarity of specific structures between languages, the LPM suggests that when learners comprehend or produce in L3, the structural representations of the two PALs are activated separately and compete with each other for linguistic proximity. Learners select the structure of the PAL with higher activation, transferring it to L3. Conversely, if learners fail to correctly parse the structure of the language during L3 input (or if L3 input is insufficient), they may activate incorrect structures more strongly, leading to non-facilitative transfer [14]. SM claims that factors, such as frequency of use, age, meta-linguistic knowledge, or literary context can also influence the activation strength [15]. Nevertheless, since they are external, both facilitative and non-facilitative influences from different PALs on the same subgrammar acquisition should be observed in the same group of learners. However, studies have shown the presence of homogeneous non-facilitative influences from PAL among learners [24,25]. A more recent study also observed homogeneous misuses in English learning among learners with native Chinese backgrounds, such as bare verbs in the subject as well as bare verbs and to-do variants in the object [28]. Thus, under the influence of some yet-unknown factors, certain circumstances may lead to a consistent misjudgment of the structural similarity of the largest influencing factor—language—by different learners, overriding the influence of other individual differences and leading to a homogeneous trend toward non-facilitative transfer within the group of learners, which contradicts the current theoretical framework of the hybrid transfer models that attribute the mechanism of non-facilitative transfer generation to individual differences only. Therefore, if the hybrid transfer models are more supported compared to the TPM, proposing a stronger explanation for the generation of non-facilitative transfer under the premise of the hybrid transfer model becomes necessary, which is the second aim of this study.

**Fig. 1:**
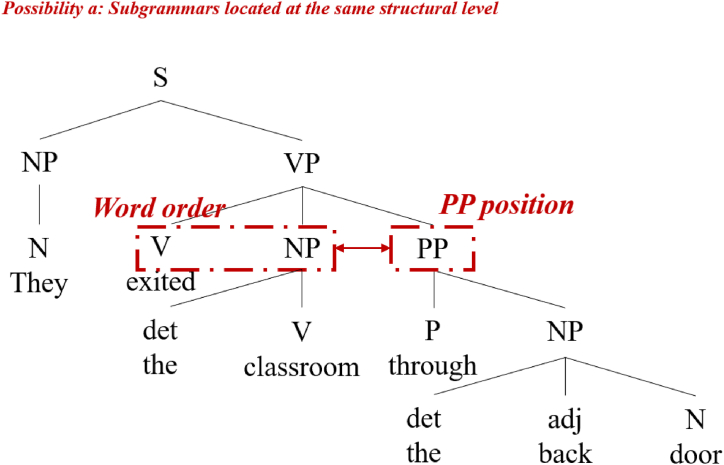
Example of relative position between PP position and WO.

Regarding the mechanism of the occurrence of non-facilitative transfers in the framework of hybrid transfer models, cognitive economy provides a possible entrance. Both TPM and hybrid transfer models assume that learners should have some degree of conscious/unconscious awareness of the target language's syntactic structure, which is the basis of transfer. These models are diverse in their understanding of the cognitive economy. According to TPM, the overall transfer is the most economical [[10], [11], [12]], whereas hybrid transfer models suggest that it may also be economical to perform small parts of subgrammatic judgments and transfers [29]. From this perspective, cognitive economy could affect learners' perceptions of subgrammars, motivating corresponding transfers in terms of the hierarchical construction of language structure. Research has demonstrated that L1 speakers prioritize structure-driven strategies and syntactic information when processing sentences, whereas L2 speakers prioritize lexical-semantic and pragmatic information [30]. Similarly, for input, L2 learners tend to prioritize real words [31]. Unlike L1, L2 and L3 are considered declarative memories [8]; thus, at least when inputting and processing L3, learners may be likely to follow a similar approach as when processing L2. Although it seems difficult to expect L3 learners to have equal acuity for every subgrammar, as SM does [15], learners are likely to take the same approach with grammar as with real words in sentences: when dealing with several units whose syntactic structures are at the same level, they are likely to prioritize the most important parts. For example, among V (verb), NP (noun phrase), and adjunct PP (pre/postposition phrase), which are governed by VP (verb phrase), the relationship between verbs and nouns, that is, the word order (WO), is most important. Even though the adjunct PP is at the same level as V and NP (Fig. 1), it can be deleted without affecting the grammatical structure and may therefore receive the least attention. Thus, although learners may understand the semantic information of the adjunct PP itself, they are less concerned about where it should be located in the VP. For learners without specialized linguistic training, the relationship between V, NP, and adjunct PP may constitute an ambiguous notion of subgrammar. Therefore, the perception of the order of V and NP will interfere with the learner's perception of the order of not only adjunct PP and V but also adjunct PP and NP. For example, native Japanese speakers whose L2 is English and L3 is Chinese judge that both languages are subject-verb-object (SVO) from the perspective of construct proximity. Their proximity judgments about the position of adjunct PP in the sentence may be directly influenced by the relative positions of V and NP located at the same structural level, and thus the positional features of adjunct PP in L2 English (post verb) to L3 Chinese (pre verb) may be incorrectly transferred. This assumption, i.e., that cognitive economy can affect learners' perceptions of subgrammars, motivating corresponding transfers in terms of the hierarchical construction of language structure, also provides a possible explanation for the mechanism of the occurrence of non-facilitative transfers in the framework of the hybrid transfer models.

### 1.2 Research objectives and implications

This article has two purposes: 1. to investigate whether the TPM and the hybrid transfer models (i.e., overall or property transfer among L3A), and 2. if the hybrid transfer models receive more support, whether the interrelationship between the position of subgrammars in sentence structure is a strong explanation for the occurrences of non-facilitative transfers, i.e., whether this factor influences the learner's judgments of interlanguage structural similarity, and hence the transfer choices in PAL. Specifically, we measured Japanese and Chinese learners' level of acquisition of WO, PP position, head position in relative clauses (RC), and interpretation of null arguments. The latter three differ from each other in terms of their position in sentence structure in relation to WO; we examined whether they are at the same level as WO, at a different level from WO, or have comprehension issues not related to sentence structure. In contrast to previous L3A studies, our experimental design used the occurrence of non-facilitative transfer as the entry point, for two main reasons: first, because, as mentioned earlier, a well-developed L3A model should be able to provide an explanation for the mechanism of non-facilitative transfer production, and the hybrid transfer models, one of our two potential candidates, are lacking a strong articulation of this part of the story; second, if we use facilitative transfer as the entry point, it is likely to be difficult to distinguish between the effects of PAL and those brought about by simply study, and it is also prone to ceiling effects; conversely, signiﬁcant misuse can help us to speciﬁcate the source of the transfer relatively unambiguously.

## 2 Methods

### 2.1 Experimental design

Puig-Mayenco et al. [32] advocate that L3 studies should use mirror designs whenever possible. However, common mirror designs (two study groups learning the same L3, where L1 and L2 are the same language but in opposite order of acquisition) have mainly been used to study the effects of the order of acquisition, e.g. using L1 Spanish → L2 English → L3 Brazilian Portuguese and L1 English → L2 Spanish → L3 Brazilian Portuguese as mirror groups of each other to examine transfer differences that arise when Spanish is used as L1 or L2 [10]. However, this traditional mirror design is unable to distinguish between models that assume that all PALs can have an effect on L3A, such as the TPM and the hybrid transfer models [14]. Therefore, through a deformed mirror-image design referencing [33], we used two experimental groups: the JEC group of L1 Japanese → L2 English → L3 Chinese and the CEJ group of L1 Chinese → L2 English → L3 Japanese, and introduced three control groups: the native Japanese group (nJ group), the native Chinese group (nC group), and the native English group learning L2 Japanese (EJ group). This design can help us confirm the effect of the difference in typological proximity/structural similarity between the languages of PAL and L3 on transfer source selection, provided that all the languages exposed are the same. We chose intermediate learners as the study population to ensure that our participants had already learned the null arguments as well as the RC and PP sentences in the target language.

### 2.2 Language features and stimuli design

#### 2.2.1 Word order (WO)

Whether linguistic genetic relationships have an impact on learners' judgments of interlanguage similarity (e.g., whether subjective similarity is higher between languages with genetic relationships compared to between two languages belonging to different language families) is unclear, and as this is not the focus of this study, to avoid the possible impact of interlanguage genetic relationships, this study was conducted on English, Japanese, and Chinese, which are languages with no linguistically significant genetic relationships. English belongs to the Indo-European language family [34], and its WO is SVO. Although Japanese's genetic relations are controversial (Koreo-Japonic relationship), its language family is considered to have no provable relations with other language families [35], and its WO is SOV. Chinese belongs to the Sino–Tibetan language family [36], and modern Mandarin Chinese is an SVO language, and the Chinese used in this study refers to Modern Mandarin Chinese. Example 1 demonstrates the WO of the same sentence in each language [37]. Additionally, another reason for choosing Japanese, English, and Chinese as the subjects of the study is that, although they belong to different language families, each of the three languages has its own similar/different aspects, which helps us to observe the characteristics of the transfer (overall or property). As for English as the L2 of the two L3 learning groups among these three languages, it is for a practical reason that Chinese and Japanese in compulsory education (elementary or junior high school) have already started to acquire English as L2 before learning Japanese/Chinese as L3.

**Table:** 

1a	John bought a book	(English: SVO)
1b	John-ga hon-o katta	(Japanese: SOV)
	John-NOM book-ACC bought	
1c	John mǎi-le yì běn shū	(Chinese: SVO)
	John buy-PERF one CL book	

Note: ACC: accusative; CL: classifier; NOM: nominative; PERF: perfective aspect.

#### 2.2.2 PP

Given the complex rules of PP position in English, Japanese, and Chinese, we set three premises when designing the stimuli. First, the choice of arguments for verbs may differ among the languages; therefore, we selected only adjunct PPs as experimental stimuli to avoid residual variables. Second, since we hypothesize that judgments of the WO structural proximity between PAL and the target L3 might influence learners’ judgments of other subgrammars at the same sentence structure level, the adjunct PP, which is directly governed by the VP, was selected as the stimulus. Moreover, since we were interested in the effect of WO on L3 PP position acquisition, all VPs in the created stimuli contained an NP. Third, in English, Japanese, and Chinese, when the adjunct PPs are in sentence-initial topic position, they appear to the left of the subject. We avoided this situation when creating stimuli, ensuring that all sentences began with the main clause subject.

Based on [38], we used three pre/postposition stimuli that did not exceed the intermediate learning level: temporal pre/postposition (Japanese: ni; Chinese: zài), locative pre/postposition (Japanese: de; Chinese: zài), and goal pre/postposition (Japanese: ni; Chinese: xiàng). In addition, because the Goal PP could sometimes be replaced by a double-object construction, all participants had to complete the sentences using pre/postposition in this study. A comparison of English, Japanese, and Chinese in the adjunct PP stimuli used in this study is summarized in Table 2. Even with a few postpositions (e.g., ago), English is a typical prepositional language with the adjunct PP located after the verb. Japanese uses case particles to identify grammatical relations, and adjunct PP is placed before the verb [39]. In Chinese, as in English, prepositions are dominant. However, the adjunct PP in Chinese only appears before the verb.

**Table 2:** Summary of adjunct PPs.

	English	Japanese	Chinese
Productive Rule of Adpositions	Preposition	Postposition	Preposition
Position of PPs	Postverbal	Preverbal	Preverbal
Syntax	NP + [Vp V + NP + PP]	NP + [Vp PP + NP + V]/NP + [Vp NP + PP + V]	NP + [Vp PP + V + NP]
Sample	Mary studies English [in the library]	Mary-ga [toshokan-de] eigo-o benkyoo-suru	Mǎlì [zài túshūguǎn] xué yīngyǔ
		Mary-NOM library-LOC English-ACC study-do	Mary in library study English

Note: ACC: accusative; LOC: locative; NOM: nominative; NP: noun phrase; PP: pre/postpositional phrase.

#### 2.2.3 RC

In Chinese and Japanese, a relative clause precedes its modifying noun, while in English, the modified noun precedes the relative clause (cf. 2). Japanese RC does not have a complementizer, while Chinese RC requires the complementizer “de” [40]. In contrast, English object-gap RC can omit a relative pronoun for the introduction of RC, while some English speakers also accept subject-gap RC without a relative pronoun [41]. To clarify the effect of PAL on learners’ L3 RC acquisition, we chose the object RC, which requires a complementizer in English. In the stimuli, English and Chinese RC require complementizers while the Japanese RC rejects them. This also avoids difficulty differences between subject-gap and object-gap RC [42]. In creating stimuli, all CPs contain a subject, verb, and object, and the number of heads that serve as subjects and objects is the same.

**Table:** 

2a	[NP the doctor [CP that Mary picked]] is John's father	(English)
2b	[NP [CP Mary-ga eranda] ishi-ha] John-no chichioya dearu	(Japanese)
	Mary-NOM picked doctor-TOP John-GEN father is	
2c	[NP [CP Mary tiāo de] yīshēng] shì John-de fùqīn	(Chinese)
	Mary pick C doctor is John's father	

#### 2.2.4 Interpretation of null arguments

English is a non-pro-drop language; in 3(a), *it* can only be interpreted as *John's sister's milk*. Japanese null arguments were previously considered *pro* [43], but recent research revealed that such null arguments should be considered as a type of Argument Ellipsis (AE) [44,45]. This is because, in addition to strict reading (cf. 4a), null arguments in Japanese also have sloppy reading (cf. 4b). In Japanese, AE can be in the position of the subject or object [46]. AE in Chinese is similarly allowed in the object position (cf. 5) [46,47]. This study only considers AE in the object position in Japanese and Chinese.

**Table:** 

3	John drank his sister's milk.	(English)
(a)	Dave also drank it (=John's sister's milk).	Strict reading
(b)	*Dave also drank it (=Dave's sister's milk).	*Sloppy reading
4	John-wa [zibun-no oneecyan-no gyunyu]-o nomimashita	(Japanese)
	John-TOP self-GEN sister-GEN milk-ACC drunk	
(a)	Dave-mo [ø (=John's sister's milk)]-o nomimashita	Strict reading
	Dave-also drunk	
(b)	Dave-mo [ø (=Dave's sister's milk)]-o nomimashita	Sloppy reading
	Dave-also drunk	
5	John hē-le [zìji-de jiějiě-de niúnǎi]	(Chinese)
	John drink-PERF self's sister's milk	
(a)	Dave yě hē-le [ø (=John's sister's milk) ]	Strict reading
	Dave also drink-PERF	
(b)	Dave yě hē-le [ø (=Dave's sister's milk) ]	Sloppy reading
	Dave also drink-PERF	

The general features of WO, PP, RC, and interpretation of null arguments for Japanese and Chinese are summarized in Table 3. When creating stimuli, each type contained only the target subgrammar and WO. For example, stimuli measuring the interpretation of null arguments contained null arguments, subjects, verbs, and objects, but not PPs and RCs.

**Table 3:** General features of subgrammars.

	Word order	Adposition		Relative clauses		Interpretation of (null) arguments
		Adjunct PP position		Complementizer		
English	SVO	Preposition	Postverbal	Relative head precedes the RC	Presence	No null arguments allowed
Japanese	SOV	Postposition	Preverbal	RC precedes its relative head	Absence	Strict and sloppy reading
Chinese	SVO	Preposition	Preverbal	RC precedes its relative head	Presence	Strict and sloppy reading

### 2.3 Predictions

Our research questions (RQs) are twofold: RQ1: Is there an overall transfer (TPM) or property transfer (the hybrid models) between L3As? RQ2: If property transfer is more supported, is the interrelationship of clauses' positions in the sentence structure a strong explanation for the mechanism by which non-facilitative transfer occurs? Based on these two RQs, we make the following predictions about the groups' performance in WO, PP, RC, and null argument explanations:

Based on the characteristics of the experimental groups, the JEC (L1 Japanese, L2 English, and L3 Chinese) group should judge English and Chinese to be similar in terms of WO and transfer L2 English to L3 Chinese, whether based on TPM or the hybrid transfer models, since they are both SVO languages. Given that PP and WO are at the same sentence structure level (cf. Fig. 1), the JEC group participants’ judgments of structural similarity of PP position would be clearly influenced by their judgments of the structural similarity of WO; namely, there is a high possibility that they would incorrectly transfer the features of postverb PP in L2 English to L3 Chinese, producing a misplacement of PP. However, for the CEJ (L1 Chinese, L2 English, and L3 Japanese) group, since L3 Japanese is the only SOV language and because there is no similarity between PAL and the target language, transfer will not occur at the level of WO; thus, the structural similarity judgments of PP location by CEJ group participants are independent of structural similarity in WO. They are, theoretically, equally likely to be positively influenced by L1 Chinese or non-facilitatively influenced by L2 English with respect to the position of PP. However, as the fundamental purpose of learning is to acquire correct knowledge, if there are no other factors that have a significant effect on the correctness of interlanguage similarity judgments, when learners are fairly confronted with an L1 that is similar to the position of PP in L3 and an L2 that is different from the position of PP in L3, it is logical for them to choose the source of transfer that can facilitate their learning, which is in this case their L1. Therefore, we hypothesized that the CEJ group was more likely to acquire facilitated transfer from L1 Chinese correctly. Since the EJ group was only influenced by L1 English, their perceptions of the PP position were theoretically unlikely to be incorrectly influenced by the structural similarity of WO, which does not exist. To sum up, the following prediction was made.

Prediction a: For Japanese learners of Chinese (JEC group), since L2 English and L3 Chinese share similar WO and PP is at the same sentence structure level as WO, it is possible that their L2 English PP knowledge will be transferred non-facilitatively to their L3 Chinese, resulting in the most PP positional misuse in tasks related to L3 Chinese production (e.g., 6, correct examples of see Table 1)., while the Chinese-speaking group learning Japanese and the native English speakers (CEJ and EJ groups) hardly produced PP positional misuse because the WO of L1 Chinese and L2 English in the CEJ group and L1 English in the EJ group were different from the WO of their target language (Japanese). Therefore, they were unlikely to produce non-facilitative transfers from PALs as a result of the interlanguage WO similarities in their learning of PP knowledge in Japanese. As a baseline, two groups of native speakers (nJ and nC) should not produce PP positional misuse.

**Table:** 

6.	*NP + [_Vp_ V + NP + PP]	（Examples of Chinese PP Misuse Influenced by Non-Facilitative Transfer from English）
	Mary xué yīngwén zài túshūshì	
	Mary study English in library	

**Fig. 2:**
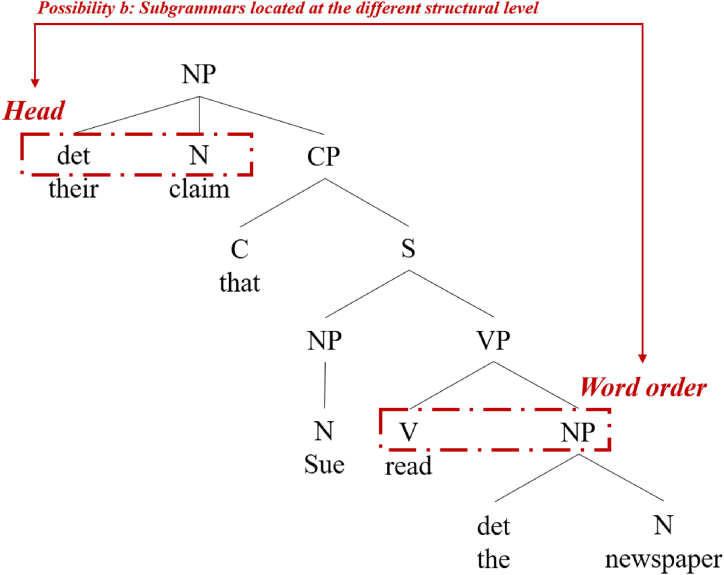
Example of relative position between RC head noun and WO.

The JEC group's judgment of the head position in RC may be less influenced by their judgment of the structural similarity of the WO, since they are at different structural levels (Fig. 2); hence, while non-facilitative transfer from L2 English could not be ruled out, it was possible for them to simultaneously and positively transfer from L1 Japanese. For the CEJ group, the positive influence from L1 Chinese created a level playing field with the non-facilitative influence from L2 English, as explained in the above predictions about PP. In this case, learners in the CEJ group could have correctly determined that their L1 Chinese was more similar to the RC structure of their L3 Japanese and avoided the non-facilitative transfer from L2 English. As for EJ, due to the inconsistent WO, for the RC head position, they may be able to determine that L1 English and L2 Japanese are not similar and reject the transfer. However, since RC is a more complex sentence structure in general, there is more potential for misuse by all learners. Therefore, we made the following prediction.

Prediction b: Although head position in RC is at a different structural level to WO, at the hypothesis stage we are not yet in a position to assert that interlanguage similarity in WO must have no effect on interlanguage similarity judgments in RC, and thus there is still a certain possibility that the JEC group may acquire non-facilitative influences from L2 English in addition to facilitative influences from L1 Japanese and produces some RC head misuse (e.g., 7, see 2c for the correct example) when completing production-related tasks. Whereas, due to the inconsistency in WO, it can be assumed that both Chinese and English native speakers learning Japanese (CEJ and EJ groups) are unlikely to affected by the non-facilitative influence of their PAL (English), and thus the CEJ and EJ groups may produce fewer or equal misuse than the JEC group. As a baseline, the two native speaker (nJ and nC) groups do not produce RC head position misuse.

**Table:** 

7.	*Head + [_CP_ C + [_TP_ NP + [_VP_ V]]]		（Examples of Chinese RC Misuse Influenced by Non-Facilitative Transfer from English）
	[yīshēng de Mary tiāo] shì John-de fùqīn		
	[doctor C Mary pick] is John's father		

Since the interpretation of null arguments is syntactically independent, the JEC group should be completely unaffected by the judgment of structural similarity of WO. Therefore, they can independently make correct judgments about the similarity between L1 Japanese and L3 Chinese, and L2 English and L3 Chinese. The same is true for the CEJ group. In contrast, since sloppy interpretation in null arguments is an understanding based entirely on context and usually not specifically mentioned in class, the EJ group may not be able to perform well in the target language (L2 Japanese) despite not recognizing any similarity between it and their native L1 English. This is corroborated by Ref. [45]. Therefore, we make the following prediction.

Prediction c: When completing comprehension-related tasks, both Japanese speakers learning Chinese (JEC group) and Chinese speakers learning Japanese (CEJ group) were more receptive to sloppy explanations, which comes from the facilitative transfer of their L1 (Japanese or Chinese). In contrast, native English speakers learning Japanese (EJ group) were more likely to reject sloppy explanations. As a baseline, both native speaker (nJ and nC) groups were receptive to sloppy explanations.

Regarding the JEC group, we can observe a non-facilitative transfer of L2 English to L3 Chinese in Prediction a. It is also possible that this non-facilitative transfer could occur in prediction b. Conversely, it is also possible that facilitated transfer from L1 Japanese to L3 Chinese could be observed in prediction c. If both predictions are verified, then the hybrid transfer models will be supported. If all three predictions are verified, the idea that the interrelationship between the position of subgrammars in sentence structure will influence learners’ transfer choice of PAL will also be supported.

### 2.4 Participants

Table 4 presents a summary of participants’ language backgrounds. All 127 participants were at least high school graduates. The sample sizes of the two L3 learning (JEC and CEJ) groups and one L2 learning (EJ) group comprised the maximum number of participants that could be recruited and tested within the time and resource constraints.

**Table 4:** Summary of participants’ language backgrounds.

		nC	JEC	nJ	EJ	CEJ
Number of participants	Men	5	11	12	9	12
	Women	22	16	14	4	16
	Non-binary	0	0	0	3	0
	Non-relevant	0	1	1	1	0
	Total	27	28	27	17	28
Age	Mean	28.48	21.25	24.58	26.44	20.29
	Mdn	28.00	21.00	22.50	27.00	20.00
	SD	4.21	1.99	7.35	7.12	1.44
Years spent studying the target language	Mean		1.98		3.81	1.84
	Mdn		2.00		3.50	2.00
	SD		0.31		1.33	0.28

The JEC and CEJ groups each comprised 28 college students from Waseda University in Japan and XI’AN International Studies University in China who were studying Chinese and Japanese. Their L2 was English. Regarding the L3 learning level, we filtered intermediate learners by the corresponding L3 test level or the number of years of study. The JEC group recruited learners who had passed Level III of the Hanyu Shuiping Kaoshi (HSK, translated as the Chinese Proficiency Test, total of 6 levels) but had not yet passed Level IV. Participants who had not taken the HSK test or who had studied Chinese for 1.5–2.5 years were also eligible. The CEJ group recruited learners who had passed the N3 level of the Japanese-Language Proficiency Test (JLPT) but had not yet passed the N2 level. The CEJ group participants had taken the JLPT exam and had an appropriate JLPT level. All participants were studying L3 in the classroom, none had lived in their L3 country for more than three months, and none had studied four or more languages. The EJ group comprised 17 adult participants from English-speaking countries. Japanese language proficiency was screened the same way as was done for the CEJ group, but because the EJ group had few eligible participants, we included some Japanese language learners with longer study periods. Because our predictions for the EJ group focused on the interpretation of null arguments, if EJ participants who had studied Japanese for a long time could not accept the sloppy reading but CEJ and JEC group learners who had studied for a relatively shorter period of time could, the effect of language background on acquisition would have been demonstrated more clearly. From this perspective, we believe that the EJ group's longer learning time caused a limited effect on this study's results. Regarding the learning method, 12, 3, and 2 participants engaged in self-study, classroom study, and immersion study (living in Japan), respectively. The two L1 control groups, nJ and nC, comprised 27 individuals each, and the language used in the test was their first language. Participation was voluntary, and all participants provided written consent prior to participating. To ensure confidentiality and anonymity of the data, participants were only identified by numbers during the experiment.

### 2.5 Stimuli

All questionnaires and tests were administered through an online survey software (Qualtrics). All words used in the stimuli were within the knowledge range of intermediate learners.

#### 2.5.1 Participant background questionnaire

The background survey section was designed with reference to Ref. [48], and included items on age, gender, final education, country of birth, country of residence, number of years of L3 study, duration of residence in a non-native country, as well as L3 study method and tested experiences. The two control groups did not receive L2-and L3-related questions. The interlingual psychotypological distance survey section was designed with reference to Ref. [49], using a paired comparison method. Participants rated subjective similarities between English and Japanese, English and Chinese, and Chinese and Japanese on a 5-point scale from 1 (completely unlike) to 5 (very similar) based on four perspectives: overall, phonology, lexicon, and syntax.

#### 2.5.2 Target language test (Mixed questionnaire with both sorting and judgment questions)

Referring to Ref. [38], we used a comprehension plus production approach in the PP and RC tasks (i.e., sorting tasks) to control for variables such as lexical choice while observing the non-facilitative influence of all PAL in L3A. This is because non-facilitative transfers are more frequent in production than in comprehension [14] and, according to Ref. [32], using the comprehension plus production task is appropriate. As the interpretation of the null arguments was purely comprehension-based, we used a judgment task to observe participants' performance. We did not design a single stimulus that contained PP, RC, and the null arguments' interpretation because it would unnecessarily lengthen the stimuli and significantly increase the participants' burden. In addition, if this were a judgment task with all three structures, it would have been necesary to ask participants to make judgments for each part of the structure of each stimulus, which would result in participants easily inferring the intent of the test. Because we wanted to see their L3 performance in a relatively natural situation, this would not have been desireable. Therefore, we designed three different tasks: two sorting tasks (involving PP and RC) and a judgment task (involving null arguments’ interpretation). They were integrated into the same language questionnaire so that they would appear randomly. Because our predictions focused mainly on intergroup cross-sectional comparisons, we deemed this design reasonable.

##### 2.5.2.1 Target language Test—PP stimuli (sorting)

**Fig. 3:**
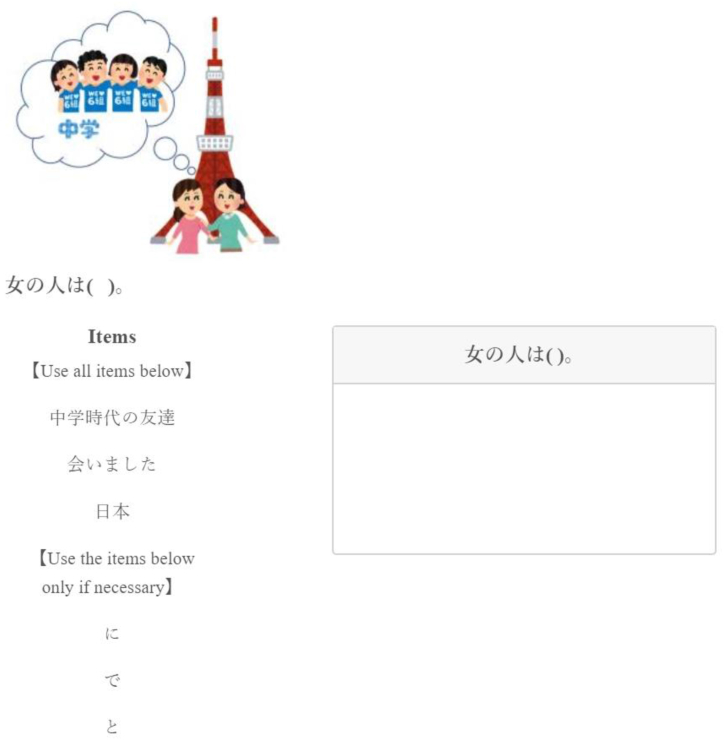
Sample of PP stimuli.

We prepared 12 stimuli, including 4 each of temporal, locative, and goal PP. A picture was presented simultaneously with the stimuli to ensure participants understood the meaning correctly. The first half of the stimuli (subject) was given, and the participants selected and completed the rest from the given items. To prevent participants from being aware of the purpose of the test, some words/phrases had to be mandatorily selected (verbs, nouns, and pre/postpositions), while some were labeled optional; among the optional choices were Japanese particle cases (to, wo, ga, etc.) or Chinese complementizers (de, le, etc.). The order of items in the “Use all items below” and “Use the items below only if necessary” categories were randomized. Participants dragged items from the left side into the answer box on the right and then adjusted their order to complete a sentence they thought was correct. Fig. 3 provides an example of PP stimuli.

##### 2.5.2.2 Target language Test—RC stimuli (sorting)

We prepared eight stimuli; for balance, the relative head noun of four stimuli was the subject, and the relative head noun of the remaining four was the object. We provided participants with pictures that corresponded to the meaning of the stimuli. RC and head were composed by participants using the given items, and the rest of the sentence was given. There were two types of items—“Use all items below” and “Use the items below only if necessary”—as in the PP stimuli. Additionally, since English, Chinese, and Japanese RC sentences differ in terms of whether complementizers are needed, “no” in the Japanese stimuli and “de” in the Chinese stimuli were placed under the category “Use the items below only if necessary” to determine whether participants would use complementizers. Items were prompted, and sorting was manipulated in ways consistent with the PP stimuli procedure.

##### 2.5.2.3 Target language Test—Null arguments stimuli (Judgment)

**Fig. 4:**
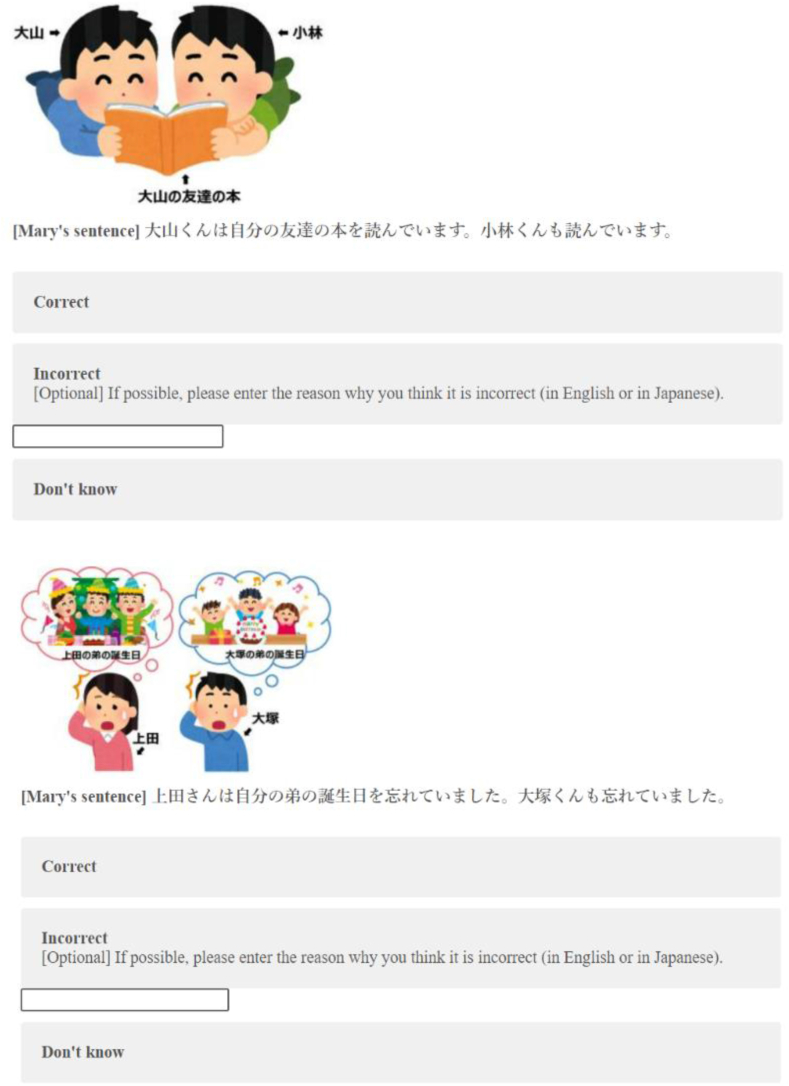
Sample of null argument stimuli (above: Strict reading; below: Sloppy reading).

We prepared 10 stimuli, 5 of which were paired with pictures for strict reading and the remaining 5 for sloppy reading. Participants were asked to judge whether the sentences were correct or incorrect based on the pictures; if incorrect, they could write down the reason for the incorrect judgment in the target or native language. Participants could choose “Don't know” if they did not understand the meaning of the stimuli sentence. Fig. 4 provides an example of null argument stimuli.

#### 2.5.3 Exclusion test

Four exclusion tests measured whether participants understood the presence of null arguments in the target language (declarative sentences in the target language containing null arguments) and whether they correctly mastered declarative sentences containing PP, RC, and a strict reading of the explicit argument *it* in L2 English. All tests were presented in the form of judgment questions, with each test containing three sentences. If learners gave incorrect or “don't know” judgments in two or more of the sentences, they were excluded from the analysis. The first test was for the JEC, CEJ, and EJ groups, and the last three tests were for the JEC and CEJ groups.

### 2.6 Procedure

At the time of application, participants were told that they were taking a survey about (foreign/native) language learning status. The actual purpose was communicated at the end of the experiment.

The formal experiment was conducted entirely online via Zoom. The participant and experimenter kept the camera and microphone on before the test began, and the participant received a link to the questionnaire via Zoom's chart function. The experimenter verbally introduced the rules while sharing the screen, showing how to complete the sorting and judgment questions with one example each.

A fictitious university student learning the participant's target language was used as a background for the test. The participant was asked to help the student with her homework (sorting) and to check whether the sentences she produced were correct (judgment). At the end of the instructions, participants were asked if they had any questions. It was made clear that if participants had questions during the test, they could ask the experimenter via Zoom. All stimuli were prompted in the target language, and other instructions were prompted in participants' native language. All sorting and judgment questions were randomly included in the same questionnaire. After completing the target language test (L3 Japanese/Chinese), both L3 groups were asked to complete an additional L2 exclusion test, in random order. Only one question was displayed on the screen at a time, and there was no time limit. If participants clicked the next question, they could not return to the previous one.

## 3 Coding and data analysis

### 3.1 Coding of PP stimuli

Four subgrammars were removed from the VPs containing adjunct PPs. Some participants occasionally forgot to use pre/postpositions in the test, because, unlike in English, pre/postpositions can sometimes be omitted in Japanese and Chinese [50,51]. Table 5 shows these omissions. Pairing these 4 subgrammars with each other resulted in 18 different patterns, in addition to the added pattern of other (incomplete entries). These 19 patterns were then grouped into three main categories based on the features of each group's target language; correct, incorrect, and other answers were further categorized into three types by misplacement pattern: WO, PP, and adposition (adP) misplacement. For coding, all answers were first assigned to the category they belonged to, resulting in the number of correct, incorrect, and other answers each. The number of answers containing the corresponding misplacement was counted separately for incorrect answers. If an answer contained different misplacement forms, it was recorded separately under the corresponding misplacement category. The number of correct answers and the number of answers containing each misplacement were used as dependent variables.

**Table 5:** The subgrammars and patterns of PP stimuli.

Subgrammar	Parameter	Order	Languages
Word order: WO	+	V O	Eng, Ch
	–	O V	Jp
PP position relative to V: VPP	+	V [PP]	Eng
	–	[PP] V	Ch, Jp
PP position relative to NP (object): OPP	+	O [PP]	Eng, Jp
	–	[PP] O	Ch, Jp
Adposition relative to NP: adP	+	preP [NP]	Eng, Ch
	–	[NP] postP	Jp
	ø	[NP]	Ch, Jp
**Pattern**		**Structure**	**Example**
1	WO+; VPP+; OPP+; adP+	V + NP + [PP preP + NP]	She [starts work at 9:00 a.m.].
2	WO+; VPP+; OPP+; adP-	V + NP + [PP NP + postP]	She [starts work 9:00 a.m. at].
3	WO+; VPP+; OPP+; adP0	V + NP + [PP NP]	She [starts work 9:00 a.m.].
4	WO+; VPP+; OPP-; adP+	V + [PP preP + NP]+NP	She [starts at 9:00 a.m. work].
5	WO+; VPP+; OPP-; adP-	V + [PP NP + postP]+NP	She [starts 9:00 a.m. at work].
6	WO+; VPP+; OPP-; adP0	V + [PP NP]+NP	She [starts 9:00 a.m. work].
7	WO+; VPP-; OPP-; adP+	[PP preP + NP]+V + NP	She [at 9:00 a.m. starts work].
8	WO+; VPP-; OPP-; adP-	[PP NP + postP]+V + NP	She [9:00 a.m. at starts work].
9	WO+; VPP-; OPP-; adP0	[PP NP]+V + NP	She [9:00 a.m. starts work].
10	WO-; VPP+; OPP+; adP+	NP + V + [PP preP + NP]	She [work starts at 9:00 a.m.].
11	WO-; VPP+; OPP+; adP-	NP + V + [PP NP + postP]	She [work starts 9:00 a.m. at].
12	WO-; VPP+; OPP+; adP0	NP + V + [PP NP]	She [work starts 9:00 a.m.].
13	WO-; VPP-; OPP+; adP+	NP + [PP preP + NP]+V	She [work at 9:00 a.m. starts].
14	WO-; VPP-; OPP+; adP-	NP + [PP NP + postP]+V	She [work 9:00 a.m. at starts].
15	WO-; VPP-; OPP+; adP0	NP + [PP NP]+V	She [work 9:00 a.m. starts].
16	WO-; VPP-; OPP-; adP+	[PP preP + NP]+NP + V	She [at 9:00 a.m. work starts]
17	WO-; VPP-; OPP-; adP-	[PP NP + postP]+NP + V	She [9:00 a.m. at work starts]
18	WO-; VPP-; OPP-; adP0	[PP NP]+NP + V	She [9:00 a.m. work starts]
19	Others	Omission of filling, etc.	

Note: Ch: Chinese; Eng: English; Jp: Japanese; NP: noun phrase; O: object; PP: prepositional/postpositional phrase; postP: postposition; preP: preposition; V: verb.

### 3.2 Coding of RC stimuli

Three subgrammars were taken from the English, Japanese, and Chinese RCs. Although we required participants to use all nouns and verbs, occasionally, they forgot to do so, resulting in RCs with subject- and object-gaps. These were also included in this analysis. Pairing 3 subgrammars with each other resulted in 12 different patterns, in addition to the added pattern of other (Table 6). The 13 patterns were grouped into three main categories based on the features of each group's target language**;** correct, incorrect, and other answers were further categorized into three types by misplacement/misuse pattern, that is, head and WO misplacement, and complementizer misuse. All answers were categorized and calculated in the same way as was the PP stimuli.

**Table 6:** The subgrammars and patterns of RC stimuli.

Subgrammar	Parameter	Order	Languages
Head position: Head	+	Head initial	Eng
	–	Head final	Ch, Jp
Word order: WO	+	V O	Eng, Ch
	–	O V	Jp
Complementizer: C	+	C	Eng, Ch
	–	Null	Jp
**Pattern**		**Structure**	**Example**
1	Head+; WO+; C+	Head + [CP C + [TP NP + [VP V + NP]]]	The claim [that John's father is a trustworthy doctor] is believable.
2	Head+; WO+; C-	Head + [CP [TP NP + [VP V + NP]]]	The claim [John's father is a trustworthy doctor] is believable.
3	Head+; WO-; C+	Head + [CP C + [TP NP + [VP NP + V]]]	The claim [that John's father a trustworthy doctor is] is believable.
4	Head+; WO-; C-	Head + [CP [TP NP + [VP NP + V]]]	The claim [John's father a trustworthy doctor is] is believable.
5	Head+; C+	Head + [CP C + [TP NP + [VP V]]]	The claim [that John's father is trustworthy] is believable.
6	Head+; C-	Head + [CP [TP NP + [VP V]]]	The claim [John's father is trustworthy] is believable.
7	Head-; WO+; C+	[CP [TP NP + [VP V + NP]]+C]+Head	[John's father is a trustworthy doctor that] the claim is believable.
8	Head-; WO+; C-	[CP [TP NP + [VP V + NP]]]+Head	[John's father is a trustworthy doctor] the claim is believable.
9	Head-; WO-; C+	[CP [TP NP + [VP NP + V]]+C]+Head	[John's father a trustworthy doctor is that] the claim is believable.
10	Head-; WO-; C-	[CP [TP NP + [VP NP + V]]]+Head	[John's father a trustworthy doctor is] the claim is believable.
11	Head-; C+	[CP [TP NP + [VP V]]+C]+Head	[John's father is trustworthy that] the claim is believable.
12	Head-; C-	[CP [TP NP + [VP V]]]+Head	[John's father is trustworthy] the claim is believable.
13	Others	Omission of filling, etc.	

Note: C: complementizer; Ch: Chinese; CP: complement phrase; Eng: English; Jp: Japanese; O: object; NP: noun phrase; TP: tense phrase; V: verb; VP: verb phrase.

### 3.3 Coding of null arguments

We counted the number of correct, incorrect, and “don't know” answers in the strict and sloppy reading stimuli. They were categorized and calculated in the same way as was the PP stimuli.

### 3.4 Data analysis

Statistical analysis was performed using SPSS 25.0 (IBM; Armonk, NY, USA). Significance was set at p < .05, with p < .001 being highly significant, and additionally, p < .1 was considered to be the presence of a favourable statistical trend. The standard test for normality indicated that all data were not normally distributed; therefore, referring to Ref. [19], a non-parametric Kruskal–Wallis H test was used for intergroup analysis; the effect size was eta-squared (*η*^*2*^), defined as small for *η*^*2*^ = 0.01, medium for *η*^*2*^ = 0.06, and large for *η*^*2*^ = 0.14 [52]. Friedman's test was used for the intragroup analysis, and Kendall's *W* provided an approximation of the effect size for the Friedman test, using Cohen's interpretation guidelines of large groups of 0.2 (small), 0.5 (moderate), and 0.8 (strong) effects [52]. The Wilcoxon signed-rank test was used for within-group comparisons of two related samples, and the Mann Whitney u test was used for intergroup comparisons of two independent samples.

## 4 Results

### 4.1 PP stimuli

**Fig. 5:**
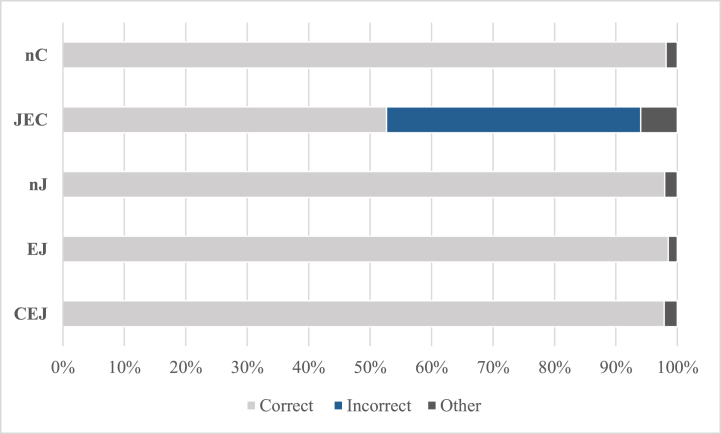
Percentage of the mean number of each answer type in the PP stimuli in the five groups.

Table 7 and Fig. 5 depict basic statistical values. A significant difference in correct answers was noticed, *H* (4) = 57.005, *p* = .000, *η*^*2*^ = 0.45. Pairwise comparisons showed that the number of correct answers was significantly smaller in the JEC group (comparison with the rest of any group: *p* = .000); no significant difference was found among the other four groups (comparison of any two groups other than the JEC group: all *p* ≥ .579). Regarding types of misplacement, a significant difference in the number of answers containing the incorrect PP position was found between the five groups, *H* (4) = 92.240, *p* = .000, *η*^*2*^ = 0.73. Pairwise comparisons showed that the JEC group had significantly more incorrect answers (comparison with any other group: *p* = .000); no difference was found among the other four groups (comparison of any two groups except the JEC group: *p* = 1.000). The JEC group produced the most PP location misplacement; the remaining four groups produced no PP location misplacement. This was consistent with Prediction a. The intergroup comparison for WO and adP misplacement was also significant (WO misplacement: *H* (4) = 38.012, *p* = .000, *η*^*2*^ = 0.30; adP misplacement: *H* (4) = 25.970, *p* = .000, *η*^*2*^ = 0.21), and the results of pairwise comparisons were consistent with PP misplacement.

**Table 7:** Mean, median, and standard deviations (SD) of the number of each answer type in the PP stimuli in the five groups.

		Correct	Incorrect				Other
			WO	PP	adP	Total	
nC Group	Mean	11.78	0.00	0.00	0.00	0.00	0.22
	Mdn	12.00	0.00	0.00	0.00	0.00	0.00
	SD	0.58	0.00	0.00	0.00	0.00	0.58
JEC Group	Mean	6.32	0.75	4.54	0.43	4.96	0.71
	Mdn	7.50	0.00	3.50	0.00	3.50	1.00
	SD	4.51	1.76	4.18	0.96	4.41	0.90
nJ Group	Mean	11.74	0.00	0.00	0.00	0.00	0.26
	Mdn	12.00	0.00	0.00	0.00	0.00	0.00
	SD	0.81	0.00	0.00	0.00	0.00	0.81
EJ Group	Mean	11.82	0.00	0.00	0.00	0.00	0.18
	Mdn	12.00	0.00	0.00	0.00	0.00	0.00
	SD	0.39	0.00	0.00	0.00	0.00	0.39
CEJ Group	Mean	11.75	0.00	0.00	0.00	0.00	0.25
	Mdn	12.00	0.00	0.00	0.00	0.00	0.00
	SD	0.44	0.00	0.00	0.00	0.00	0.44

Note*: adP: adposition misplacement; PP: pre/postposition phrase misplacement; WO: word-order misplacement*.

### 4.2 RC stimuli

**Fig. 6:**
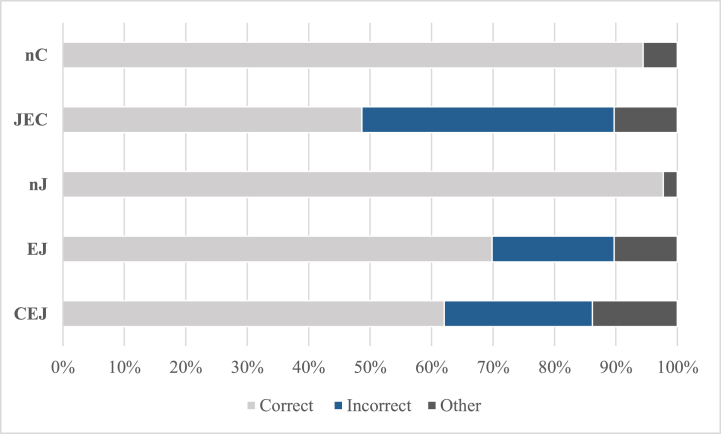
Percentage of the mean number of each answer type in the RC stimuli in the five groups.

Table 8 and Fig. 6 depict basic statistical values. A significant difference in correct answers was noticed, *H* (4) = 56.428, *p* = .000, *η*^*2*^ = 0.45. Pairwise comparisons revealed that the number of correct answers did not differ significantly between the EJ and CEJ and between nJ and nC groups (all *p* ≥ .451), whereas a significant tendency was observed between the JEC and EJ (*p* = .093) and the JEC and CEJ (*p* = .076) groups. Significant differences were found between each of the three language learning (JEC, EJ, CEJ) and two native (nC, nJ) groups (all *p* ≤ .002). The data showed a significant difference in the number of answers containing the incorrect head position, *H* (4) = 44.903, *p* = .000, *η*^*2*^ = 0.36. Pairwise comparisons showed that the JEC group had significantly more incorrect answers than did the other four groups (comparison with any other group: *p* = .000); no difference was found between the other four groups (comparison of any two groups except the JEC group: all *p* ≥ .292). The JEC group produced the most misuses of RC head positions; there was no significant difference between the remaining four groups in the misuse of this structure, consistent with Prediction b. The intergroup comparison for WO misplacement was significant, *H* (4) = 50.221, *p* = .000, *η*^*2*^ = 0.40, and the results of pairwise comparisons were consistent with head misplacement. Significant differences in complementizer misuse between groups were observed, *H* (4) = 63.320, *p* = .000, *η*^*2*^ = 0.50. Pairwise comparisons revealed that the number of answers containing complementizer misuse did not differ significantly between any two of the three language learning groups (all *p* ≥ .105) and no significant difference was found between the nC and nJ groups (*p* = 1.000). However, there were significant differences between nC/nJ and the three language learning groups (*p* = .000).

**Table 8:** Mean, median, and SD of the number of each answer type in the RC stimuli in the five groups.

		Correct	Incorrect				Other
			Head	WO	C	Total	
nC Group	Mean	7.56	0.00	0.00	0.00	0.00	0.44
	Mdn	8.00	0.00	0.00	0.00	0.00	0.00
	SD	0.75	0.00	0.00	0.00	0.00	0.75
JEC Group	Mean	3.89	1.61	0.64	2.29	3.29	0.82
	Mdn	3.50	1.00	0.50	2.00	3.50	0.50
	SD	2.41	1.99	0.78	1.65	2.11	1.06
nJ Group	Mean	7.81	0.00	0.00	0.00	0.00	0.19
	Mdn	8.00	0.00	0.00	0.00	0.00	0.00
	SD	0.40	0.00	0.00	0.00	0.00	0.40
EJ Group	Mean	5.59	0.35	0.00	1.53	1.59	0.82
	Mdn	6.00	0.00	0.00	1.00	1.00	1.00
	SD	2.24	1.06	0.00	1.74	1.87	0.95
CEJ Group	Mean	4.96	0.18	0.04	1.82	1.93	1.11
	Mdn	5.00	0.00	0.00	1.00	1.00	0.00
	SD	2.77	0.55	0.19	2.04	2.04	1.59

*Note: C: complementizer misuse; Head: head misplacement; WO: word-order misplacement*.

### 4.3 Null arguments stimuli

**Fig. 7:**
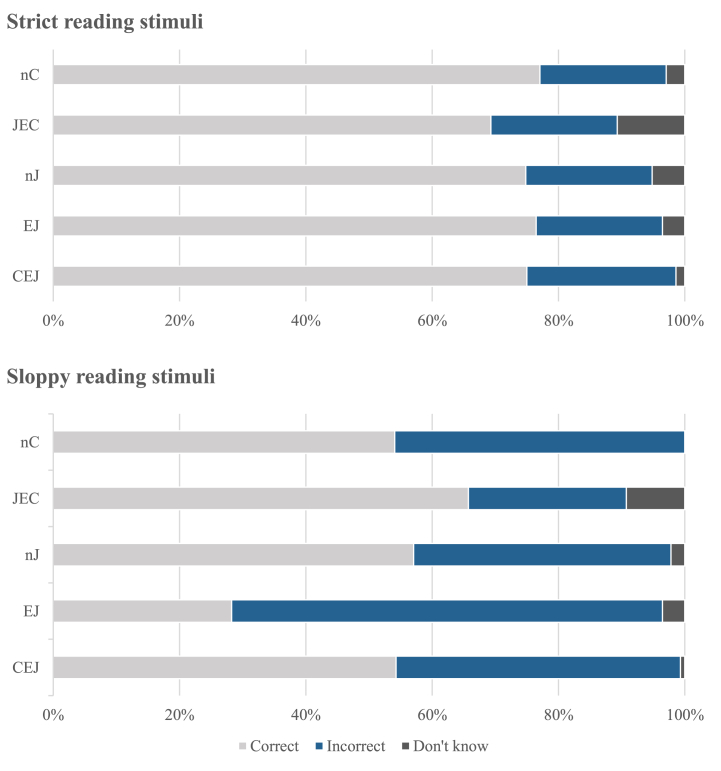
Percentage of the mean number of each answer type in the null arguments stimuli in the five groups.

Table 9 and Fig. 7 depict basic statistical values. Regarding the strict reading stimuli, there were no significant differences between groups in the number of correct answers, *H* (4) = 0.831, *p* = .934, *η*^*2*^ = 0.01. However, regarding the sloppy reading stimuli, a significant difference was found, *H* (4) = 11.505, *p* = .021, *η*^*2*^ = 0.09. Pairwise comparisons revealed that there were significant differences between EJ and CEJ (*p* = .018), EJ and nJ (*p* = .011), EJ and JEC (*p* = .001), and EJ and nC (*p* = .018) groups. The rest of the groups were not significantly different from each other (all *p* ≥ 274). Thus, regarding sloppy reading, the acceptance of EJ was significantly smaller than in the other four groups, consistent with Prediction c.

**Table 9:** Mean, median, and SD of the number of each answer type in the null arguments stimuli in the five groups.

		Correct	Incorrect	Don't know
**nC Group**				
Strict reading stimuli	Mean	3.85	1.00	0.15
	Mdn	4.00	1.00	0.00
	SD	1.13	1.18	0.46
Sloppy reading stimuli	Mean	2.70	2.30	0.00
	Mdn	2.00	3.00	0.00
	SD	1.88	1.88	0.00
**JEC Group**				
Strict reading stimuli	Mean	3.46	1.00	0.54
	Mdn	4.00	0.50	0.00
	SD	1.55	1.44	0.88
Sloppy reading stimuli	Mean	3.29	1.25	0.46
	Mdn	4.00	1.00	0.00
	SD	1.63	1.43	0.69
**nJ Group**				
Strict reading stimuli	Mean	3.74	1.00	0.26
	Mdn	4.00	1.00	0.00
	SD	1.43	1.39	0.81
Sloppy reading stimuli	Mean	2.85	2.04	0.11
	Mdn	3.00	2.00	0.00
	SD	1.73	1.72	0.32
**EJ Group**				
Strict reading stimuli	Mean	3.82	1.00	0.18
	Mdn	4.00	1.00	0.00
	SD	1.33	1.27	0.53
Sloppy reading stimuli	Mean	1.41	3.41	0.18
	Mdn	1.00	4.00	0.00
	SD	1.54	1.70	0.53
**CEJ Group**				
Strict reading stimuli	Mean	3.75	1.18	0.07
	Mdn	4.00	1.00	0.00
	SD	1.21	1.25	0.26
Sloppy reading stimuli	Mean	2.71	2.25	0.04
	Mdn	2.00	2.50	0.00
	SD	1.88	1.88	0.19

### 4.4 Within-group comparison of misplacement/misuse

**Fig. 8:**
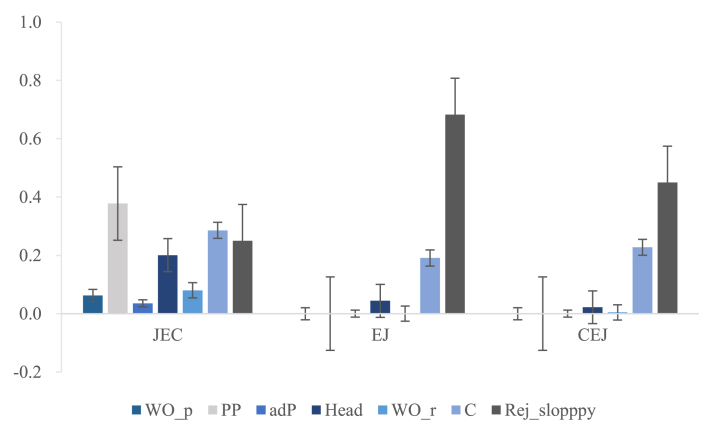
Mean of the Percentage of Answers Containing Each Type of Misplacement/Misuse in the Three Stimuli Note: adP: percentage of answers containing adP misplacement; C: percentage of answers containing complementizer misuse; Head: percentage of answers containing head misplacement; PP: percentage of answers containing pre/postposition phrase misplacement; Rej_sloppy: percentage of the number of answers that reject sloppy explanations; WO_p: percentage of answers containing word order misplacement in PP stimuli; WO_r: percentage of answers containing word order misplacement in RC stimuli.

We conducted a within-group comparison for JEC, EJ, and CEJ groups. Considering the total number of each type of stimuli differed, we calculated the percentage of each type of misplacement/misuse by dividing the number of answers containing each type of misplacement/misuse by the total number of questions for the corresponding stimuli. Table 10 and Fig. 8 depict basic statistical values.

**Table 10:** Mean, median, and SD of the percentage of answers containing each type of misplacement/misuse in the three stimuli.

		PP stimuli			RC stimuli			Rej_slopppy
		WO_p	PP	adP	Head	WO_r	C	
JEC Group	Mean	0.06	0.38	0.04	0.20	0.08	0.29	0.25
	Mdn	0.00	0.29	0.00	0.13	0.06	0.25	0.20
	SD	0.15	0.35	0.08	0.25	0.10	0.21	0.29
EJ Group	Mean	0.00	0.00	0.00	0.04	0.00	0.19	0.68
	Mdn	0.00	0.00	0.00	0.00	0.00	0.13	0.80
	SD	0.00	0.00	0.00	0.13	0.00	0.22	0.34
CEJ Group	Mean	0.00	0.00	0.00	0.02	0.00	0.23	0.45
	Mdn	0.00	0.00	0.00	0.00	0.00	0.13	0.50
	SD	0.00	0.00	0.00	0.07	0.02	0.25	0.38

*Note: adP: percentage of answers containing adP misplacement; C: percentage of answers containing complementizer misuse; Head: percentage of answers containing head misplacement; PP: percentage of answers containing pre/postposition phrase misplacement; Rej_sloppy: percentage of the number of answers that reject sloppy explanations; WO_p: percentage of answers containing word order misplacement in PP stimuli; WO_r: percentage of answers containing word order misplacement in RC stimuli*.

Regarding the JEC group, the data showed a significant difference among the percentages of the number of answers containing different types of misplacement/misuse, *χ*^2^ (6) = 54.653, *p* = .000, *W* = 0.325. Pairwise comparisons revealed that while the percentage of answers containing pre/postposition phrase misplacement was not significantly different from ones containing complementizer misuse (*p* = .688). However, they had a favourable statistical trend higher than the percentage of answers that rejected sloppy explanations (*p* = .063) and significantly higher than the percentage of answers containing head misplacement (*p* = .026), WO misplacement in RC stimuli (p = .000), WO misplacement in PP stimuli (*p* = .000), and adP misplacement (*p* = .000). The percentage of answers containing complementizer misuse was not significantly different from that reject sloppy explanations (*p* = .146), but had a significant tendency to be higher than the percentage of answers containing head misplacement (*p* = .068), and was significantly higher than WO misplacement in RC stimuli (*p* = .001), WO misplacement in PP stimuli (*p* = .000), and adP misplacement (*p* = .000). The percentage of the number of answers that reject sloppy explanations did not significantly different from that containing head misplacement (*p* = .711), but had a significant tendency to be higher than the percentage of the number of answers that included the WO misplacement in RC stimuli (*p* = .059), and was significantly higher than WO misplacement in PP stimuli (*p* = .009), and adP misplacement (*p* = .001). The percentage of answers containing head misplacement was not significantly different from that containing WO misplacement in RC stimuli (*p* = .130) but was significantly higher than the percentage of answers containing WO misplacement in PP stimuli (*p* = .024) and adP misplacement (*p* = .005). No significant differences were found between the percentage of answers containing WO misplacement in RC and PP stimuli and the percentage of answers containing adP misplacement (all *p* ≥ .194).

Regarding the EJ group, the data showed a significant difference among the percentages of the number of answers containing different types of misplacement/misuse, *χ*^2^ (6) = 75.898, *p* = .000, *W* = 0.744. Pairwise comparisons showed that the percentage of the number of answers that reject sloppy explanations was significantly higher than all the remaining types of misplacement/misuse (all *p* ≤ .035). The percentage of answers containing complementizer misuse was significantly higher than all types of misplacement/misuse (all *p* ≤ .035) except the percentage of the number of answers that reject sloppy explanations. No significant differences were found between the percentage of answers containing pre/postposition phrase misplacement, WO misplacement in PP and RC stimuli, adP misplacement, and head misplacement (all *p* ≥ .691).

Regarding the CEJ group, the data showed a significant difference among the percentages of the number of answers containing different types of misplacement/misuse, *χ*^2^ (6) = 95.189, *p* = .000, *W* = 0.567. Pairwise comparisons showed that the percentage of the number of answers that reject sloppy explanations was not significantly different from those containing complementizer misuse (*p* = .477) but significantly higher than all the remaining types of misplacement/misuse (all *p* = .000). The percentage of answers containing complementizer misuse was significantly higher than all types of misplacement/misuse (all *p* ≤ .002) except the percentage of the number of answers that reject sloppy explanations. No significant differences were found between the percentage of answers containing pre/postposition phrase misplacement, WO misplacement in PP and RC stimuli, adP misplacement, and head misplacement (all *p* ≥ .458).

### 4.5 Comparison of interlingual psychotypological distance between two L3 learning groups

**Fig. 9:**
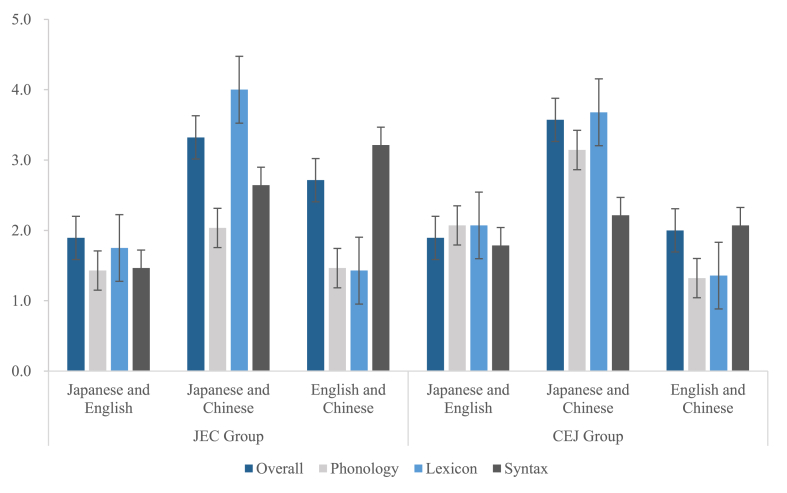
Mean of interlingual psychotypological distance for JEC and CEJ groups.

Table 11 and Fig. 9 depict basic statistical values. The CEJ group's judgments of similarity between Japanese and English phonology (*U* = 218.500, *z* = −2.910, *p* = .004, *η*^*2*^ = 0.16) and between Japanese and Chinese phonology (*U* = 171.000, *z* = −3.606, *p* = .000, *η*^*2*^ = 0.24)) were significantly higher than those of the JEC group, while the JEC group made significantly higher judgments than did the CEJ group for overall (*U* = 246.500, *z* = −2.333, *p* = .020, *η*^*2*^ = 0.10) and syntax (*U* = 181.000, *z* = −3.417, *p* = .001, *η*^*2*^ = 0.22) similarity between English and Chinese; no significant differences were found in the rest of the intergroup comparisons (all *p* ≥ .101).

**Table 11:** Mean, median, and SD of interlingual psychotypological distance for JEC and CEJ groups.

		JEC			CEJ			
		Japanese and English	Japanese and Chinese	English and Chinese	Japanese and English	Japanese and Chinese	English and Chinese	
Overall	Mean	1.89	3.32	2.71	1.89	3.57	2.00	
	Mdn	2.00	3.00	3.00	2.00	4.00	2.00	
	SD	0.83	0.90	1.08	0.74	0.96	0.77	
Phonology	Mean	1.43	2.04	1.46	2.07	3.14	1.32	
	Mdn	1.00	2.00	1.00	2.00	3.00	1.00	
	SD	0.69	1.00	0.74	0.94	0.93	0.55	
Lexicon	Mean	1.75	4.00	1.43	2.07	3.68	1.36	
	Mdn	1.00	4.00	1.00	2.00	4.00	1.00	
	SD	0.97	0.86	0.63	1.02	0.86	0.73	
Syntax	Mean	1.46	2.64	3.21	1.79	2.21	2.07	
	Mdn	1.00	3.00	3.00	2.00	2.00	2.00	
	SD	0.69	1.13	1.20	0.96	0.92	1.02

Within-group comparisons for the JEC group revealed significant differences between paired comparisons of interlingual psychotypological distances for the three languages in all four perspectives (overall: *χ*^2^ (2) = 26.467, *p* = .000, *W* = 0.473; phonology: *χ*^2^ (2) = 7.547, *p* = .023, *W* = 0.135; lexicon: *χ*^2^ (2) = 36.731, *p* = .000, *W* = 0.680; syntax: *χ*^2^ (2) = 25.531, *p* = .000, *W* = 0.456). The JEC group considered the distance between Japanese and Chinese to be the closest in terms of overall distance (all *p* ≤ .027), followed by a closer distance from English to Chinese than to Japanese (*p* = .016). Regarding phonology, the JEC group considered the distance from Japanese to Chinese to be closer than that to English (*p* = .033). There was a tendency for significant differences between Chinese-to-Japanese and Chinese-to-English distances (*p* = .095), while English-to-Chinese distances were not significantly different from Japanese-to-English distances (*p* = .640). Regarding lexicon, the JEC group considered the distance between Japanese and Chinese to be the closest (all *p* = .000), with no significant difference between English and the other two languages (*p* = .504). Finally, with regard to syntax, the JEC group considered no significant distances between Chinese and the other two languages (*p* = .161), although the distance between Japanese and English was the furthest (all *p* ≤ .001).

Regarding the CEJ group, there were significant differences between the paired comparisons of interlingual psychotypological distances for the three languages in all four perspectives (overall: *χ*^2^ (2) = 32.345, *p* = .000, *W* = 0.599; phonology: *χ*^2^ (2) = 39.747, *p* = .000, *W* = 0.736; lexicon: *χ*^2^ (2) = 46.083, *p* = .000, *W* = 0.823; syntax: *χ*^2^ (2) = 6.000, *p* = .050, *W* = 0.111). The CEJ group identified the overall linguistic distance between Japanese and Chinese as the closest (all *p* = .000), finding no significant difference in distance between English and the other two languages (*p* = .540). Regarding phonology, the CEJ group considered the distance between Japanese and Chinese to be the closest (all *p* ≤ .002), followed by the distance from English to Japanese being more closer than that to Chinese (*p* = .012). Regarding lexicon, the CEJ group considered the linguistic distance between Japanese and Chinese to be the closest (all *p* = .000), finding no significant difference between English and the other two languages (*p* = .134). Finally, with regard to syntax, the CEJ group found a tendency for significant differences between Japanese-to-Chinese distances and Japanese-to-English distances (*p* = .066), whereas there was no significant difference between distances between Japanese-to-Chinese and English-to-Chinese (*p* = .221). Furthermore, there was no significant difference between discourse distances between English-to-Chinese and those between Japanese-to-English (*p* = .540).

## 5 Discussion

### 5.1 PP stimuli

No influence of PAL on the other two language learning groups was found except for JEC, for whom PP misplacement was consistent with our prediction, supporting the hypothesis that the interrelationship between the positions of subgrammars in sentence structure is an influential factor in PAL transfer selection. Because the languages differed, another reason for only the JEC group showing the influence of PAL is that PP knowledge is more difficult in Chinese than in Japanese. However, if subgrammatical positions have no effect, then the JEC group's transfer choice for PAL in the PP stimulus should be random. Thus, because there could be facilitative and non-facilitative influences from L1 Japanese and L2 English, the JEC group should have the same probability of obtaining a facilitative influence from L1 Japanese in the PP stimuli as in the RC stimuli (correct head position), which is inconsistent with our results. Therefore, the JEC group showed that the PP position representation in L3 was clearly affected by the non-facilitative influence of L2 English because PP is at the same sentence structure level as WO; learners' judgments of its structural similarity were disturbed by the structural similarity of the dominant subgrammar WO (VO in both Chinese and English, OV in Japanese).

The within-group comparison of misplacement/misuse also showed that adP misplacement was significantly less than was PP misplacement. One possibility is that adP and WO are not on the same sentence structure level, so learners’ judgments of their similarity are not affected by WO. However, similar performance to adP misplacement should be observed in head misplacement and complementizer misuse, which are also not on the same sentence structure level as WO, although the current data do not support this. A second possibility is that since both English and Chinese are prepositions-dominant, when JEC learners transfer WO from English (L2) to Chinese (L3), the adP is transferred together with the PP position, under the influence of cognitive economy. Then it can be speculated that WO, as the dominant subgrammar, can similarly influence other subgrammars at different structural levels. That is, not only the use of adP, but also the similarity of WO should have the same influence on the judgments of head position and complementizer presence/absence in the JEC group, giving rise to an apparent non-facilitative and facilitative transfer from L2 English (head-initial/complementizer presence) to L3 Chinese (head-final/complementizer absence); however, the data do not support this.

### 5.2 RC stimuli

Effects from PAL were observed in the JEC, EJ, and CEJ groups, with the JEC group having more head misplacement than others, in line with our prediction. As for complementizer misuse, there was no significant difference between the three language learning groups, probably because RC complementizers are neither at the same structural level as the dominant subgrammar WO, as PP, nor contain pragmatic information, as the head. Words that are, or may be misidentified as, complementizers (“de” in Chinese, “no” in Japanese) are usually introduced to learners for the first time as a marker of possession, rather than being combined with NP to form a PP as a whole, as in the case of adP. Thus, RC complementizers may have been relatively unnoticed knowledge for learners in the three groups, thus spawning more transfer. This would explain why equally significant complementizer misuse occurred in the three language learning groups and why no significant difference in the JEC group was observed between complementizer misuse and PP misplacement. The EJ group had no choice but to non-facilitatively transfer their native language when unsure whether they should use complementizers. Since both Chinese and English have complementizers, either transfer would result in a non-facilitative effect for the CEJ group.

### 5.3 Null arguments stimuli

Regarding the sloppy interpretation of the null arguments stimuli, the EJ group's rejection was higher than were those of all other groups, in line with our prediction. Although there is no AE in English, the VP ellipsis (VE) phenomenon exists. For example, 6 can have two strict interpretations—that Perry and David polished Perry's car together and that *his* here refers to an individual not mentioned in the sentence. However, 6 can also have a sloppy interpretation—Perry and David polished their own cars separately. EJ participants without specialized linguistic knowledge may have focused on the similarity of the surface structure of the sentences (repeated use of the same verbs in sentences 3–4, when learning L2 Japanese), thus transferring the strict interpretation represented by 3 rather than VE, which also allows sloppy reading. This is supported by the results of [45]. Regarding the EJ group's performance, the higher acceptance of the null arguments sloppy reading in the CEJ and JEC groups can be presumed to result from a facilitated transfer from their L1 (Japanese or Chinese) rather than from a transfer of VE in their shared L2, English.

6. Perry polished his car and David did too.

### 5.4 Within-group comparison of misplacement/misuse

The within-group comparison of misplacement/misuse showed that JEC and CEJ had relatively high rejection rates for sloppy reading. This could be attributed to the different processes of interpretation and production. Except for the null arguments judgment task, the stimuli used in this study were presented as a comprehension-plus-production approach. Although intertwined [53], the processes are two distinct mechanisms [54]. Thus, comparing sloppy reading rejection rates directly to other misplacement/misuse incidences may be inappropriate**.** This could also be attributed to the way we set up the stimuli. We described the judgment question as “Mary freely created Japanese sentences based on the picture provided. Please judge whether the sentences created by Mary are correct or not.” However, different participants may have different benchmarks for correctness or incorrectness, especially with stimuli that contain ambiguous expressions. The criteria for judging correctness of the stimuli include being grammatically correct, not contradicting the situation in the diagram based on grammatical correctness, expressing the situation in the diagram precisely and unambiguously based on grammatical correctness, or having a natural expression (to clarify the affiliation, we added “Japanese: jibun-no/Chinese: ziji-de” in all stimuli to emphasize “own,” which is unnecessary or even unnatural in daily Japanese or Chinese expressions, as pointed out by some participants when they explained why they judged the stimuli incorrectly). A large proportion of participants who judged the stimuli to be incorrect filled in only the sentences they thought were correct, or filled nothing at all, leaving us with no way to determine why they rejected these sloppy reading stimuli.

### 5.5 Comparison of interlingual psychotypological distance between two L3 learning groups

The CEJ group seemed more inclined to perceive the pronunciation of Japanese as being closer to both English and to Chinese than did the JEC group, perhaps because Japanese has both Chinese-derived kanji and English-based kana words (apple, when expressed in Japanese with kanji as “林檎” is pronounced “ringo,” which is similar to the Chinese pronunciation of apple “pingguo.” When expressed in English-based kana words, it can be written as “アップル” and pronounced “appuru,” similar to the English word “apple”). Moreover, the JEC group, both overall and grammatically, perceived English and Chinese as being closer compared to the CEJ group, consistent with our experimental findings that the JEC group was clearly influenced by L2 English both in the PP and RC stimuli. Overall, although the data showed that the JEC group was more aware of the similarity between English and Chinese than was the CEJ group, they were also as fully aware of the similarity between Japanese and Chinese as was the CEJ group. The TPM model asserts that psychotypological proximity is the most powerful factor influencing the transfer of PAL in L3A [32] and, according to Rothman [12], learners are likely to judge psychotypological proximity in the order of Lexicon → Phonological/Phonotactic Cues → Functional Morphology → Syntactic Structure. However, we have not uncovered evidence that the JEC group is likely to obtain a holistic perception of similarity between English and Chinese in terms of psychotypology compared to Japanese and Chinese. The hybrid transfer model, represented by the LPM, suggests that the role of overall psychotypological proximity should decrease with increasing exposure to L3 while that of structural similarity should increase [14]. From this perspective, the performance of the JEC group across the three stimuli provides more support for the claim that structural proximity, rather than overall psychotypological proximity, is an important influencing factor for transfer (at least in the intermediate stages of learning). The coexistence of the representations of L1 and L2 that we observed in L3 only became possible because the learners’ similarity judgments for PAL and the target language were not holistic but property-by-property based on structural similarity.

Overall, factors such as differences in ease of acquisition of different grammars, or differences in ease of acquisition of the same grammar across languages, as well as common grammatical features of the two PALs do pose some challenges in explaining our non-facilitative transfer source. However, in the result discussion of each of the subgrammars in this section, we have explained in detail the phenomena that the above factors can lead to and compared them with our data. With the exception of the CEJ group's inability to specify the transfer source of the complement misuse in generating RCs (because both their L1 Chinese and L2 English require the use of complements to connect RCs and its relative head), the possibilities posed by the other factors mentioned above have been largely ruled out. Thus, at least in the JEC group, we observed both non-facilitative influences (complementizer misuse) and facilitative transfer (sloppy reading of the null arguments stimuli) from L1 Japanese, and non-facilitative influences from L2 English (PP misplacement and head misplacement). If the TPM is supported, we should observe the overall transfer of only one of the PALs (L1 Japanese or L2 English) in the JEC group, e.g., when the JEC group decides to transfer their L1 Japanese, they should transfer the Japanese feature of placing the PP before the verb without producing a misplacement of the type of placing the PP after the verb as it is done in English; on the other hand, if the JEC group decides to transfer their L2 English, then when interpreting the null arguments, they should reject sloppy interpretation as the EJ group did. Thus, our results support the hybrid transfer models, i.e., the transfer from PAL to L3 is partial.

Meanwhile, our data also oppose the hypothesis of Ortin and Fernandez-Florez [55] that the L2SF and TPM models complement each other. The TPM argues that psychotypological proximity is the decisive force driving the selection of transferable systems, whereas the L2SF argues that in the absence of psychotypological proximity, L2 is the preferred language system that can be transferred to the developing grammar of L3. However, even with no psychotypological influence, we do not see an advantage from L2 English in the CEJ group. In general, structural similarity played a greater role than did typological proximity.

On the other hand, since neither the LPM nor the SM specifically describe how the similarity of the selected structures was assessed and according to which criterion “recruitment factors for L1 or L2 grammars were weighted” [56], this study verified whether the interrelationship between the position of subgrammars in sentence structure influences transfer selection of PAL. Our results were consistent with our predictions, supporting the hypotheses. However, while the interrelationship between the positions of subgrammars may be influential, it is certainly not the only relationship. Our results highlight several other potential factors, such as whether the target subgrammar contains pragmatic information and how the target subgrammar is acquired. The rule seems to be related to the uneven distribution of attention.

The rule seems to be that subgrammars that are easily ignored are more likely to induce the need for transfer. That is, uneven distribution of attention during input for easily ignored subgrammars/structures, etc. → very weak target representation → need to transfer PAL → interlingual structural similarity selection and transfer guided by cognitive economy → facilitative/non-facilitative influences. If this inference is correct, then it is necessary to explore the role that attention plays in PAL transfer selection in L3, as learners’ attention to different structures is likely to influence their judgments of similarity across structures and lead to different transfer strategies.

Regarding attention, one possible measure is to refer to SLA. According to the theory of SLA, attention plays a pivotal role in the acquisition process of INPUT-INTAKE-INTERNAL SYSTEM-OUTPUT [57] and is considered a prerequisite for acquisition [58,59]. Since the greatest difference between L3A and SLA centers on the presence of plural transfer sources in L3A, there is no reason to assume that attention does not play a similar role in other parts of L3A as it does in SLA. The selection for plural PAL in L3A is not a separate cognitive process, but part of the overall L3A process. In contrast to studies that have mostly focused on the transfer characteristics of PAL, our study also creates an improved understanding of the cognitive economy, which is not merely conceptual, e.g., merely subjectively speculating that a certain state of affairs seems to be more economical, which can lead to different models declaring that their claims are in line with this notion, but pragmatic, as our study gives the so-called cognitive economy a range of applicability in the hypothesizing process. Thus, the interrelationship between the positions of subgrammars (in the same or different sentence structure level) may be affected by cognitive economy, and the results of our experiments support the hypothesis that learners' judgments of inter-subgrammatical similarity are indeed likely to be influenced by their judgments of interlanguage subjective similarity to other subgrammars that are in the same level of sentence structure. This research approach could help inform the experimental design of other future studies on the concept of cognitive economy. And, we also believe that attention, as a representation of cognitive economy, should be discussed more often, even preferentially, in future L3A studies. For example, it is important to understand whether the non-facilitative transfer observed in the present study, caused by the interrelationship between the position of subgrammars in sentence structure, is induced by the uneven distribution of attention during input (i.e., the above inference), or if the problem occurs during intake, which may require an eye-movement study for verification. Further, if uneven distribution of attention is indeed one of the main culprits of PAL non-facilitative transfer, it is important to determine the factors, other than the interrelationship between the position of subgrammars in sentence structure, that contribute to the uneven distribution of attention. This would not be limited only to theoretical studies of L3A but would also have implications for practical teaching and learning.

Finally, given that the results of the present study support transfer from both L1 and L2, there is also the question of how we should explain the mechanism of transfer. A convincing explanation is proposed by the theory of multiple grammars (MG), which argues that when learning a new language, instead of changing grammatical (mainly morphosyntactic) rules in response to new input, learners should create parallel sets of rules [18]. According to MG, all the learner needs to do when learning L2, L3, and even Ln is to add new rules/features to the grammar base and reassess their productivity. Then, the so-called transfer in L3A is simply a judgment and choice made by the learner for different parameters that are activated simultaneously [14]. Similarly, the effect of relative positional relations among subgrammars on the cognitive economy can be explained in terms of activation levels. For example, for the JEC group, since both L2 English and L3 Chinese are more productive in WO with the VO parameter, the activation of the latter in English drives the activation of the PP feature that is more productive in English (the postverb parameter, which is at the same syntactic structure level). In short, due to the cognitive economy, the interrelationship between the positions of subgrammars becomes an important factor that influences the level of activation and, thus, the transfer. However, L1 is maintained by procedural memory, whereas L2, L3, or Ln are maintained by declarative memory [8]. These types of memory are neurolinguistically distinct [60,61]. However, if L1 and other acquired languages do not belong to the same memory source, how should we interpret the MG? The interpretation of the activation levels of the subgrammar parameters from a neurolinguistic perspective may be an important topic for future studies.

A limitation of this study is that participants may have different criteria for judging what is correct in null arguments stimuli. This needs to be clarified in future research by allowing participants to give more reasons as to why they judged a stimuli to be incorrect (grammatical misuse, ambiguity, unnaturalness, etc.). In addition, learners at the intermediate level were chosen as the subjects of the current study. However, the scope of the model's application was not investigated, which will be addressed in future studies.

## 6 Conclusion

This study aimed to answer two questions: First, is the transfer from PAL to L3A overall or partial? Second, if the transfer is partial, considering that the influencing factors are structural similarity and the perspective of cognitive economy, can the interrelationship between the position of subgrammars in sentence structure influence learners’ judgments on structural similarity between languages, thereby affecting transfer selection? We conducted a mirror-image design test (L1 Japanese - L2 English - L3 Chinese learners and L1 Chinese - L2 English - L3 Japanese learners), focusing on PP position in declarative sentences, head position in RCs, and sloppy reading of null arguments. Regarding the first question, we observed both L1 and L2 representations in the L3 of the JEC group, supporting the hybrid transfer models represented by LPM (that transfer occurs property-by-property) and arguing against the model that advocates overall transfer (TPM). In response to the second question, our data show that, at least for intermediate L3 learners, the structural similarity is a more powerful factor in explaining PAL transfer in L3/Ln acquisition than is typological proximity.

## Funding

This work was supported by 10.13039/501100004423Waseda University.

## Ethics declarations

In order to ensure that the experimental process fully respects the human rights and dignity of the participants and strictly protects their personal information, this study was reviewed and approved by the Ethics Review Committee on Research with Human Participants of Waseda University, with the approval number: no. 2021-256. All participants provided informed consent to participate in the study. All participants provided informed consent for the publication of their task/questionnaire data.

## CRediT authorship contribution statement

**Xiaoyu Luan:** Conceptualization, Data curation, Formal analysis, Funding acquisition, Methodology, Project administration, Writing – original draft, Writing – review & editing. **Masakazu kuno:** Conceptualization, Formal analysis, Supervision. **Ayaka Sugawara:** Conceptualization, Methodology, Supervision. **Yayoi Kawasaki:** Formal analysis, Methodology, Supervision. **Eriko Sugimori:** Conceptualization, Data curation, Methodology, Supervision, Writing – original draft, Writing – review & editing.

## Declaration of competing interest

The authors declare that they have no known competing financial interests or personal relationships that could have appeared to influence the work reported in this paper.
