# Supplementary material for: The effect of previously acquired languages on third language acquisition
Source: Heliyon. 2024 Feb 14;10(4):e26202. doi: 10.1016/j.heliyon.2024.e26202 (PMC10882039; doi:10.1016/j.heliyon.2024.e26202)
Supplement: Multimedia component 1 [file mmc1.pdf]

Study Material 2: Stimuli List for the Target Language Test and Exclusion Test

Type of Stimuli		Number of questions	Japanese Stimuli	Chinese Stimuli	*English Translation*
PP (Sorting)	Temporal	4	彼女は大学時代に中国語を勉強しました。	她在大学时代学了汉语。	*She studied Chinese in her college days.*
			女の人は午後6時に映画館へ到着しました。	女人在下午6点到了电影院。	*The woman arrived at the cinema at 6pm.*
			息子が15歳の時に犬を拾いました。	儿子在15岁的时候捡了一只狗。	*My son picked up a dog at age 15.*
			彼は昨年12月に家を買いました。	他在去年12月买了房。	*He bought the house in December last year.*
	Locative	4	妹は家で音楽を聴いています。	妹妹在家听音乐。	*My sister is listening to music at home.*
			女の人は日本で中学時代の友達と会いました。	女人在日本遇到了初中同学。	*The woman met her junior high school classmate in Japan.*
			彼は喫茶店でこの本を書きました。	他在咖啡馆写了这本书。	*He wrote the book in a cafe.*
			弟が映画館で映画を見ています。	弟弟在电影院看电影。	*My brother is watching a movie at the cinema.*
	Goal	4	鈴木くんは先生に一つの質問をしました。	铃木同学向老师问了一个问题。	*Suzuki-san asked a question to the teacher.*
			男の人は友達に結婚のことを話しました。	男人对朋友说了结婚的事。	*The man spoke about the marriage to his friend.*
			男の人は上司に申請書を出しました。	男人向上司提交了申请。	*The man submitted the application to his supervisor.*
			店員さんは私にありがとうと言いました。	店员对我说了"谢谢"。	*The clerk said "thank you" to me.*
RC (Sorting)	Head as Subject	4	ママとパパが結婚式を挙げるあの日は金曜日でした。	妈妈和爸爸举办婚礼的那天是星期五。	*The day that Mom and Dad had their wedding was a Friday.*
			彼がお酒を買っていたお店が閉店しました。	他买酒的店关门了。	*The store where he bought the wine was closed.*
			彼女が歌を歌う声がとてもやさしいです。	她唱歌的声音很温柔。	*The voice that she singing the song is very soft.*
			娘は絵を描くスピードがとても早いです。	女儿画画的速度很快。	*The speed that my daughter paint pictures is very fast.*
	Head as Object	4	彼は猫が牛乳を飲む動画を見ています。	他正在看小猫喝牛奶的动画。	*He is watching a video that a cat is drinking milk.*
			彼は娘さんがピアノを習っている学校へ行きました。	他去了女儿学钢琴的学校。	*He went to the school where his daughter was learning piano.*
			小林さんは息子に仕事が見つからなことを心配しています。	小林先生担心儿子找不到工作的事情。	*Mr. Kobayashi was worried about the fact that his son could not find a job.*
			山田くんは妹が日記を書いていたノートを見ました。	山田同学看了他妹妹写日记的笔记本。	*Yamada-san reads the notebook where his sister keeps her diary.*

Interpretation of Null Arguments (Judgment)	Strict Reading	5	大山くんは自分の友達の本を読んでいます。小林くんも読んでいます。	大山同学正在看自己的朋友的书，小林同学也正在看。	*Oyama-san is reading his own friend's book. Kobayashi-san also reading ø.*
			石井さんは自分の担任の先生の授業を受けました。中村さんも受けました。	石井同学上了自己的老师的课，中村同学也上了。	*Ms. Ishii took a class from her own homeroom teacher. Ms. Nakamura also took ø.*
			高橋さんは自分の家の近くの神社に行きました。佐藤さんも行きました。	高桥先生去了自己家附近的神社，佐藤小姐也去了。	*Mr. Takahashi went to a shrine near his own home. Ms. Sato also went ø.*
			鈴木さんは自分のお兄さんの結婚式に参加しました。森下さんも参加しました。	铃木同学参加了自己的哥哥的婚礼。森下同学也参加了。	*Mr. Suzuki attended his own brother's wedding. Mr. Morishita also attended ø.*
			坂本さんは自分の友達の家に行きました。西田くんも行きました。	坂本同学去了自己的朋友家。西田同学也去了。	*Sakamoto-san went to his own friend's house. Nishida also went ø.*
	Sloppy reading	5	村山くんは自分のお姉ちゃんの牛乳を飲みました。田中くんも飲みました。	村山同学喝了自己的姐姐的牛奶。田中同学也喝了。	*Murayama-kun drank his own sister's milk. Tanaka-kun also drank ø.*
			松本さんは自分の犬の写真を撮っています。佐野さんも撮っています。	松本女士正在拍自己的狗的照片，佐野先生也正在拍。	*Matsumoto-san is shooting his own dog's picture. Mr. Sano is also shooting ø.*
			北村さんは自分の車を修理しました。杉本さんも修理しました。	北村先生修理了自己的车，杉本小姐也修理了。	*Kitamura-san repaired his own car. Sugimoto-san also repaired ø.*
			山下さんは自分の家のお風呂場を掃除しました。藤川さんも掃除しました。	山下先生打扫了自己家的浴室，藤川小姐也打扫了。	*Yamashita-san cleaned his own bathroom. Fujikawa-san also cleaned ø.*
			上田さんは自分の弟の誕生日を忘れていました。大塚くんも忘れていました。	上田小姐忘记了自己的弟弟的生日，大塚先生也忘记了。	*Ueda-san forgot her own brother's birthday. Otsuka-san also forgot ø.*
	Understanding the presence of null arguments in the target language	3	娘は自分のメガネを壊してしまったが、お父さんが直しました。	女儿弄坏了自己的眼镜，但她爸爸修好了。	*The daughter broke her glasses, but her father fixed ø.*
			男の子は本を買いましたが、まだ読み終わっていません。	男孩买了一本书，但还没有看完。	*The boy bought a book, but hasn't finished ø.*
			ウサギは温泉が好きです。クマも好きです。	兔子喜欢泡温泉，熊也喜欢。	*The rabbit loves the hot springs, and the bear loves ø too.*
	**English Stimuli**				
	Mastery of PP in English	3	Mary went to school by bus.		
			John starts work at 9:00 a.m.		

**Exclusion Test (Judgment)**			Mr. White wrote a letter to his daughter.
	Mastery of RC in English	3	They claim that Sue knew the woman.<br>Jim realized that David ate up the last bit of cake.<br>Kate assumed that Robert disliked the book.
	Understanding the strict reading of explicit argument *it* in English	3	John saw the picture of him mother. Mary saw it, too.<br>Mary believes that her paper will be accepted, and John also believes that it will be accepted.<br>Mary wiped her car, and John wiped it, as well.

Note. Items shown in bold blue in the PP and RC stimuli are the parts where participants were asked to sort.
