# Supplementary material for: The effect of previously acquired languages on third language acquisition
Source: Heliyon. 2024 Feb 14;10(4):e26202. doi: 10.1016/j.heliyon.2024.e26202 (PMC10882039; doi:10.1016/j.heliyon.2024.e26202)
Supplement: Multimedia component 2 [file mmc2.pdf]

[Participation Application Form] A Survey of Japanese Language Learning for Native English Speakers

[Survey Introduction]

■Content: Japanese language test

■Payment: **15 USD (or approximately 1, 500 yen)**, by bank transfer or Amazon E-gift Card

■Measure of participation: **Online questionnaire survey**

■Cautions:

1. Since each question in this questionnaire is accompanied by a picture, we recommend you take the test in a place with a relatively stable network environment.
2. Please take the test in a quiet environment where you can concentrate.
3. The test can be completed on a smartphone, but we strongly recommend you use a computer or tablet to complete the test.

[Application Requirements]

1. Native English speaker
2. **Have experience in learning Japanese** (about 1.5~ 3 years of study or N3~4)

We will contact you by e-mail within a week of your application.

For more information, please contact
[blinded for review]

Press the "→" button to display the next page.

→

1.Name

First Name

Last Name

2.Student ID

（Only if you are a student of [blinded for review]）

3.Email address

4.Age

5.Gender

- Male
- Female
- Non-binary
- Non-relevant

6.Degree

Graduate school (Doctor)

Graduate school (Master)

College (Bachelor)

High School

Other

7.Country of origin

8.Country of residence

Press the "→" button to display the next page.

←

→

9.Indicate your native language(s) and any other languages you have studied or learned, the age at which you started using/studying each language, and the total number of years you have spent using/studying each language.

***Notes**

For "Age to start using/studying", please indicate the age when you started using/studying the language, e.g., 3rd year of elementary school, etc.

For "Years of use/study", you may have learned a language, stopped using it, and then started using it again. Please give the total number of years. If you do not know the exact number, you can enter an approximate number; if it is less than one year, you can use decimals (e.g., 1.5 years).

Language	Age to start using/studying*	Years of use/study*

10.If you have lived or traveled in countries other than your country of residence for three months or more, then indicate the name of the country, your length of stay (in Months), the language you used, and the frequency of your use of the language for each country.

***You may have been to the country on multiple occasions, each for a different length of time. Add all the trips together.**

***Frequency of use: 1. Never; 2. Sometimes; 3. Regularly; 4. Often; 5. Always**

Country	Length of stay (in Months)*	Language	Frequency of use*

11.Indicate the way you learned or acquired your non-native language(s). Check one or more boxes that apply.

***<u>Immersion</u>: e.g., Immigrating to another country where the dominant language is different from your native language so you learn this language through immersion in the language environment**

	Immersion*	Classroom instruction	Self-learning	Other
Japanese	☐	☐	☐	☐
Other1 [ ]	☐	☐	☐	☐
Other2 [ ]	☐	☐	☐	☐

Press the "→" button to display the next page.

← →

12.Rate your current ability in each of the languages you have studied or learned (including the native language).

	1: Very poor	2	3	4	5: Excellent
English	○	○	○	○	○
Japanese	○	○	○	○	○
Other1	○	○	○	○	○
Other2	○	○	○	○	○

13.If you have taken any standardized language proficiency tests (e.g., TOEFL, IELTS, HSK, etc.), then indicate the name of the test, the language assessed, and the score or level you received for each. If you do not remember the exact score, then indicate an "Approximate score" instead.

Language	Test	Year taken	Score/Level

← →

We thank you for your time spent taking this survey.
Your response has been recorded.
