# Supplementary material for: The effect of previously acquired languages on third language acquisition
Source: Heliyon. 2024 Feb 14;10(4):e26202. doi: 10.1016/j.heliyon.2024.e26202 (PMC10882039; doi:10.1016/j.heliyon.2024.e26202)
Supplement: Multimedia component 3 [file mmc3.pdf]

. **Participant Number**

.

# Japanese language test

## Test Introduction

**■Since each question in this questionnaire is accompanied by a picture, we recommend you take the test in a place with a relatively stable network environment.**
**■There are 33 questions in total. There is no time limit for each question.**
**■Please answer all questions by yourself. We will not test your individual ability or rank you higher or lower. The purpose is to see the overall trend of correctness or incorrectness. Please do not be so concerned about the correct answers that you consult others, use dictionaries or search engines, etc., as this will distort the data. After you have completed the answer, press the "→" button to display the next question. Once you press "→", you cannot go back to the previous question.**

## Test Background and Contents

**Mary is a university student studying Japanese. Please help her with her Japanese homework. There are two types of questions in the homework.**

### [Type 1: Judgment]

**Mary freely created Japanese sentences based on the picture provided. Please judge whether the sentences created by Mary are correct or not.**
**If you think it is wrong, please write a Japanese sentence that you think is correct if possible or give us your reason in your native laguage (this is optional).**

### [Type 2: Sorting]

**Please help Mary with a sorting problem that she is not good at. Based on the picture provided, select the necessary choices in order from the items on the left side, and put them in the answer column on the right side in correct order to form the sentence.**
**You can move an item on the left side by long-pressing and putting it in the answer column.**
**If you put a wrong answer in the answer column, you can move it back to the left side by long-pressing it.**
**If you want to adjust the order of the items in the answer column, long-press and hold the item you want to change the order.**

**We will now present one example each of [Type 1: Judgment] and [Type 2: Sorting]. The answers to the example questions will not be counted as results. Please click the "→" button in the lower right corner to proceed to the example.**

## . [Type 1: Judgment] Example Question

**Determine if the Japanese sentence Mary created based on the picture presented is correct.**

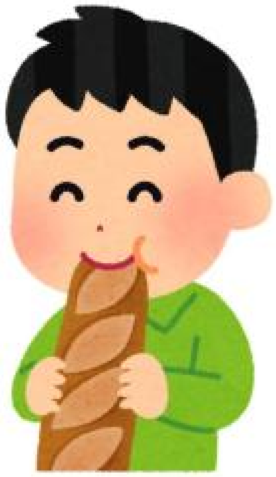

**[Mary's sentence] 男の子はパンを食べています。**

- ◯ **Correct**
- ◯ **Incorrect**
  [Optional] If possible, please enter the reason why you think it is incorrect.
- ◯ **Don't know**

.

## [Type 2: Sorting] Example Question

**Based on the picture provided, select the necessary choices in order from the items on the left side, and put them in the answer column on the right side in correct order to form the sentence.**

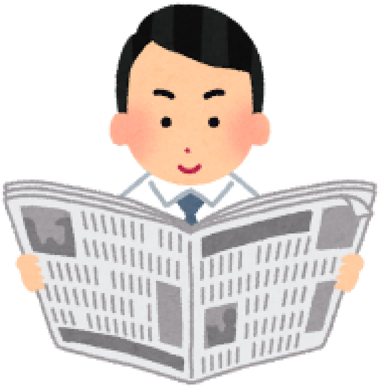

**男の人は(　)。**

| 項目 | 男の人は ( )。 |
|---|---|
| 【Use all items below】 | |
| 新聞紙 | |
| 読んでいます | |
| 【Use the items below only if necessary】 | |
| を | |
| に | |

.

**Are you confident in your answer?**

| 1.Not confident at all | 2.Not very confident | 3.Somewhat confident | 4.Very confident |
|---|---|---|---|
| ◯ | ◯ | ◯ | ◯ |

. **Press the "→" button in the lower right corner to proceed to the test.**

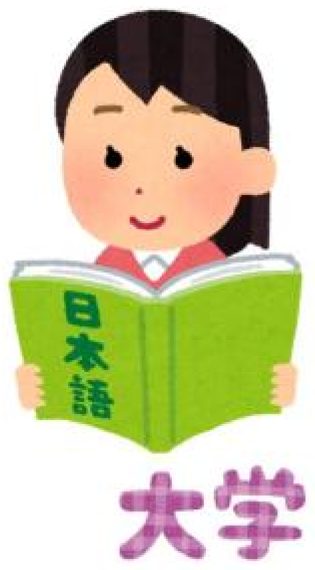


.
彼女は(　　)。

| 項目 | |
|---|---|
| 【Use all items below】<br>大学時代<br>勉強しました<br>日本語<br>【Use the items below only if necessary】<br>で<br>に<br>を | **彼女は( )。** |

. Are you confident in your answer?

| 1.Not confident at all | 2.Not very confident | 3.Somewhat confident | 4.Very confident |
|---|---|---|---|
| ○ | ○ | ○ | ○ |

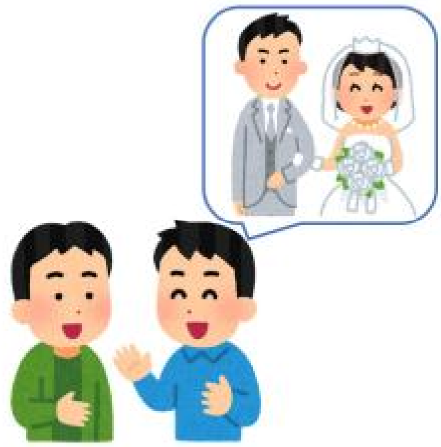

.
男の人は(　)。

| 項目 | 男の人は( )。 |
| --- | --- |
| 【Use all items below】<br>友達<br>話しています<br>結婚のこと<br>【Use the items below only if necessary】<br>が<br>に<br>を | |

. Are you confident in your answer?

| 1.Not confident at all | 2.Not very confident | 3.Somewhat confident | 4.Very confident |
| --- | --- | --- | --- |
| ◯ | ◯ | ◯ | ◯ |

.

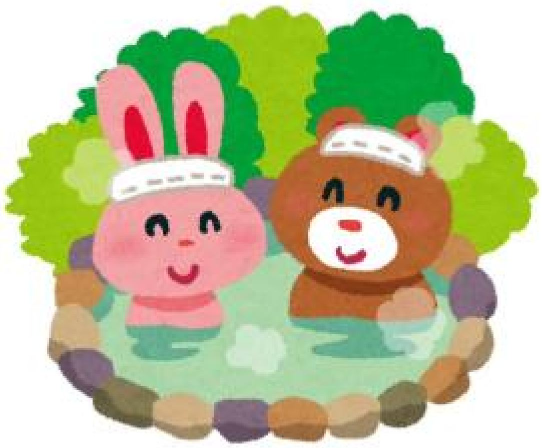

**[Mary's sentence] ウサギは温泉が好きです。クマも好きです。**

◯ **Correct**

◯ **Incorrect**
[Optional] If possible, please enter the reason why you think it is incorrect.

◯ **Don't know**

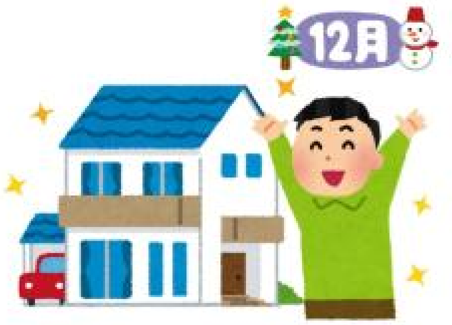


.
彼は(　　)。

| 項目 | 彼は( )。 |
|---|---|
| 【Use all items below】<br>買いました<br>去年12月<br>家<br>【Use the items below only if necessary】<br>を<br>で<br>に | |

. Are you confident in your answer?

| 1.Not confident at all | 2.Not very confident | 3.Somewhat confident | 4.Very confident |
|---|---|---|---|
| ◯ | ◯ | ◯ | ◯ |

.

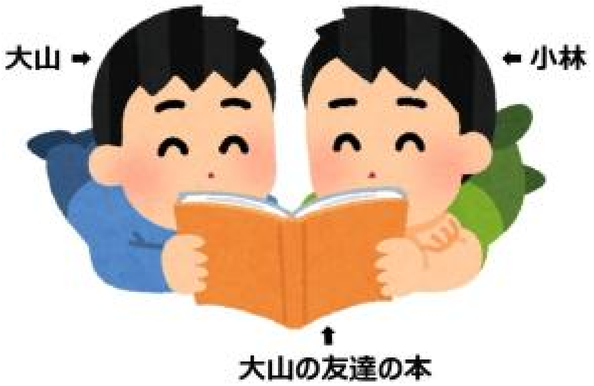


**[Mary's sentence] 大山くんは自分の友達の本を読んでいます。小林くんも読んでいます。**

◯ **Correct**

◯ **Incorrect**

[Optional] If possible, please enter the reason why you think it is incorrect.

◯ **Don't know**

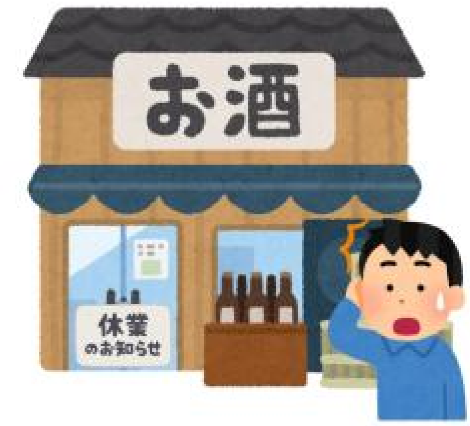


.

(　)が閉店しました。

**項目**

【Use all items below】

お酒

買っていた

彼

お店

【Use the items below only if necessary】

の

を

が

**( )が閉店しました。**

. Are you confident in your answer?

1.Not confident at all ◯ 2.Not very confident ◯ 3.Somewhat confident ◯ 4.Very confident ◯

.

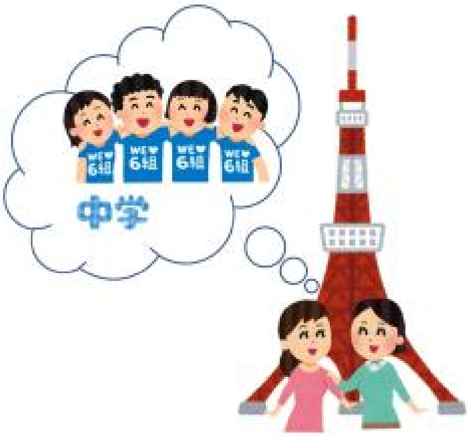


**女の人は(　)。**

| 項目 | 女の人は( )。 |
| --- | --- |
| 【Use all items below】<br>中学時代の友達<br>会いました<br>日本<br>【Use the items below only if necessary】<br>に<br>で<br>と | |

. Are you confident in your answer?

| 1.Not confident at all | 2.Not very confident | 3.Somewhat confident | 4.Very confident |
| --- | --- | --- | --- |
| ◯ | ◯ | ◯ | ◯ |

.

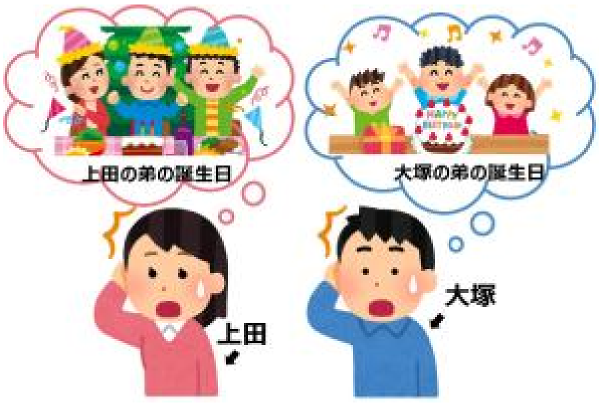


**[Mary's sentence] 上田さんは自分の弟の誕生日を忘れていました。大塚くんも忘れていました。**

- ◯ **Correct**
- ◯ **Incorrect**
  [Optional] If possible, please enter the reason why you think it is incorrect. [ ]
- ◯ **Don't know**

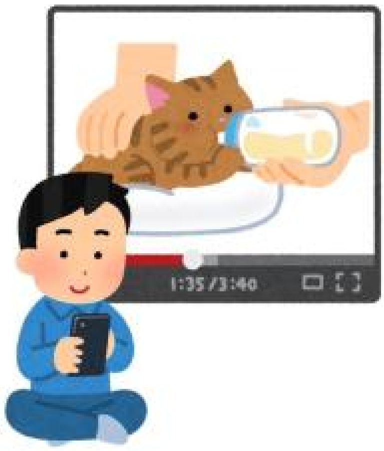


.
彼は(　)を見ています。

**项目**

【Use all items below】

牛乳

飲む

動画

猫

【Use the items below only if necessary】

を

が

の

**彼は( )を見ています。**

. Are you confident in your answer?

| 1.Not confident at all | 2.Not very confident | 3.Somewhat confident | 4.Very confident |
|---|---|---|---|
| ◯ | ◯ | ◯ | ◯ |

.

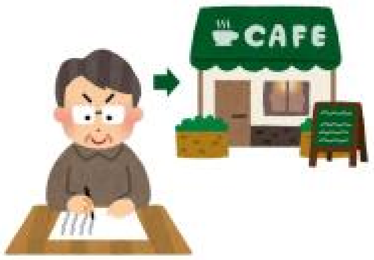


彼は(　)。

| 項目 | 彼は( )。 |
| --- | --- |
| 【Use all items below】<br>この本<br>喫茶店<br>書きました<br>【Use the items below only if necessary】<br>を<br>で<br>に | |

. Are you confident in your answer?

| 1.Not confident at all | 2.Not very confident | 3.Somewhat confident | 4.Very confident |
| --- | --- | --- | --- |
| ◯ | ◯ | ◯ | ◯ |

.

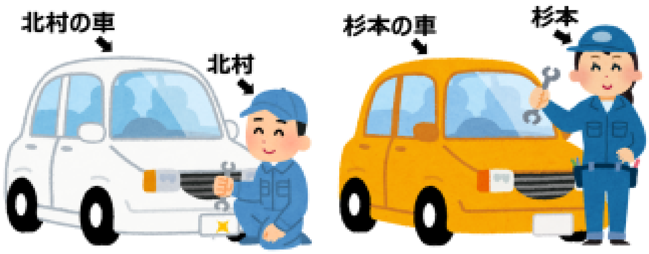


**[Mary's sentence]北村さんは自分の車を修理しました。杉本さんも修理しました。**

- ◯ **Correct**
- ◯ **Incorrect**
  [Optional] If possible, please enter the reason why you think it is incorrect.
- ◯ **Don't know**

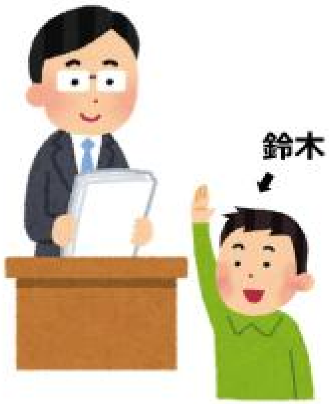


.

鈴木くんは(　)。

**項目**

【Use all items below】

1つの質問

先生

しました

【Use the items below only if necessary】

を

と

に

**鈴木くんは( )。**

. Are you confident in your answer?

| 1.Not confident at all | 2.Not very confident | 3.Somewhat confident | 4.Very confident |
| --- | --- | --- | --- |
| ○ | ○ | ○ | ○ |

.

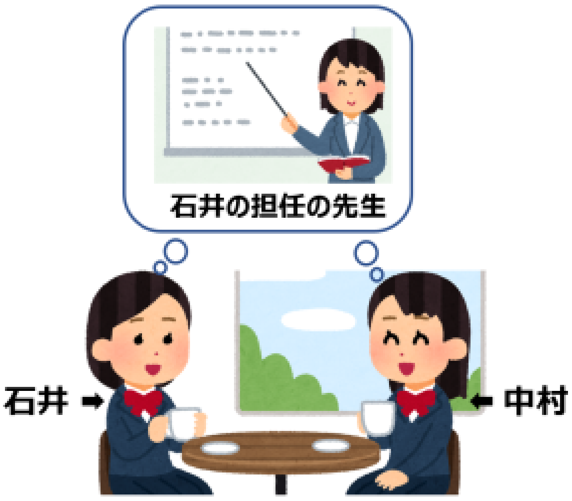


**[Mary's sentence] 石井さんは自分の担任の先生の授業を受けました。中村さんも受けました。**

- ○ **Correct**
- ○ **Incorrect**
  [Optional] If possible, please enter the reason why you think it is incorrect.
- ○ **Don't know**

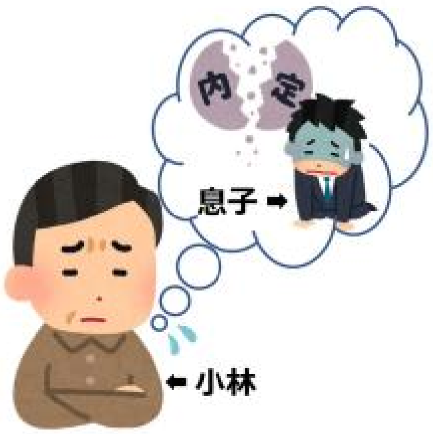


.
小林さんは( )を心配しています。

| 項目 | 小林さんは( )を心配しています。 |
| --- | --- |
| 【Use all items below】<br>息子<br>こと<br>見つからない<br>仕事<br>【Use the items below only if necessary】<br>の<br>を<br>が | |

. Are you confident in your answer?

1.Not confident at all ○ 2.Not very confident ○ 3.Somewhat confident ○ 4.Very confident ○

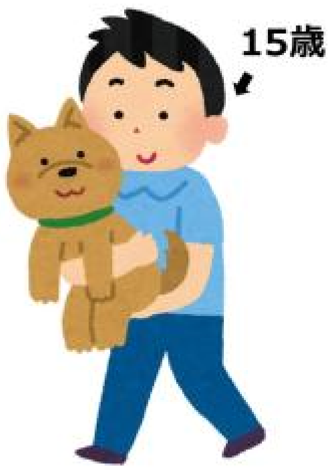


.
息子が( )。

| 項目 | |
|---|---|
| 【Use all items below】<br>15歳の時<br>拾いました<br>犬<br>【Use the items below only if necessary】<br>に<br>を<br>で | 息子が( )。 |

. Are you confident in your answer?

1.Not confident at all ◯ 2.Not very confident ◯ 3.Somewhat confident ◯ 4.Very confident ◯

.

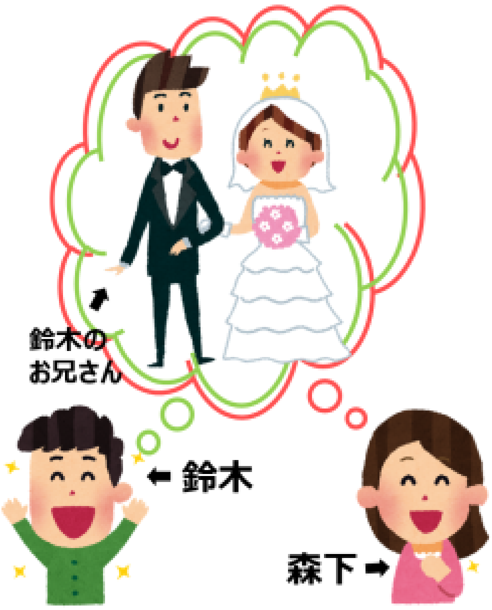


**[Mary's sentence]鈴木さんは自分のお兄さんの結婚式に参加しました。森下さんも参加しました。**

◯ **Correct**

◯ **Incorrect**

[Optional] If possible, please enter the reason why you think it is incorrect.

◯ **Don't know**

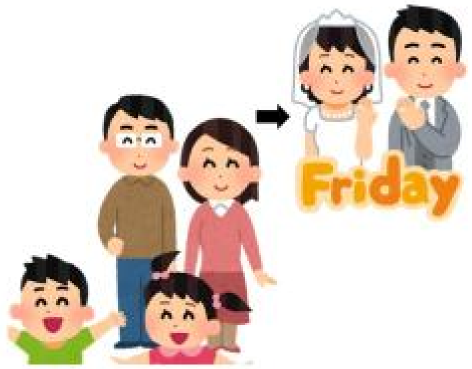


.
(　)は金曜日でした。

| 項目 | ( )は金曜日でした。 |
| --- | --- |
| 【Use all items below】<br>ママ<br>結婚した<br>パパ<br>あの日<br>【Use the items below only if necessary】<br>と<br>が<br>の | |

. Are you confident in your answer?

| 1.Not confident at all | 2.Not very confident | 3.Somewhat confident | 4.Very confident |
| --- | --- | --- | --- |
| ◯ | ◯ | ◯ | ◯ |

.

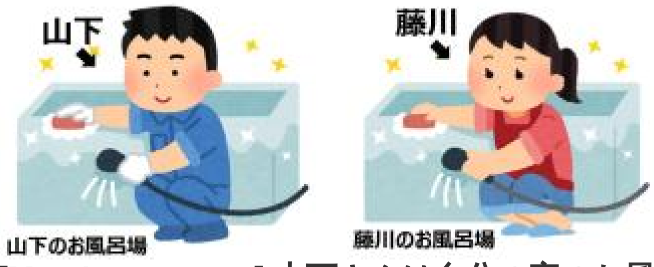


**[Mary's sentence] 山下さんは自分の家のお風呂場を掃除しました。藤川さんも掃除しました。**

◯ **Correct**

◯ **Incorrect**

[Optional] If possible, please enter the reason why you think it is incorrect. [　　　　　]

◯ **Don't know**

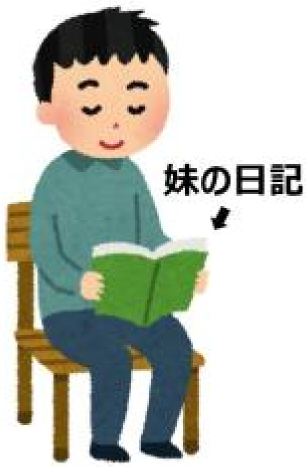


.
彼は(　)を読んでいます。

| 項目 | 彼は( )を読んでいます。 |
|---|---|
| 【Use all items below】<br>日記<br>ノート<br>妹<br>書いていた<br>【Use the items below only if necessary】<br>を<br>の<br>が | |

. Are you confident in your answer?

| 1.Not confident at all | 2.Not very confident | 3.Somewhat confident | 4.Very confident |
|---|---|---|---|
| ○ | ○ | ○ | ○ |

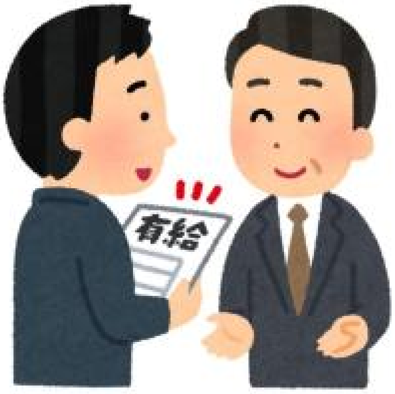


.
男の人は(　)。

| 項目 | 男の人は( )。 |
| --- | --- |
| 【Use all items below】<br>上司<br>出しました<br>申請書<br>【Use the items below only if necessary】<br>へ<br>に<br>を | |

. Are you confident in your answer?

1.Not confident at all ◯ 2.Not very confident ◯ 3.Somewhat confident ◯ 4.Very confident ◯

.

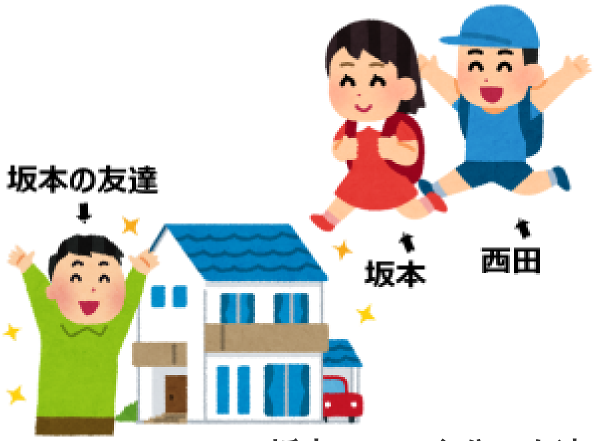


**[Mary's sentence]坂本さんは自分の友達の家に行きました。西田くんも行きました。**

- ◯ **Correct**
- ◯ **Incorrect**
  [Optional] If possible, please enter the reason why you think it is incorrect.
- ◯ **Don't know**

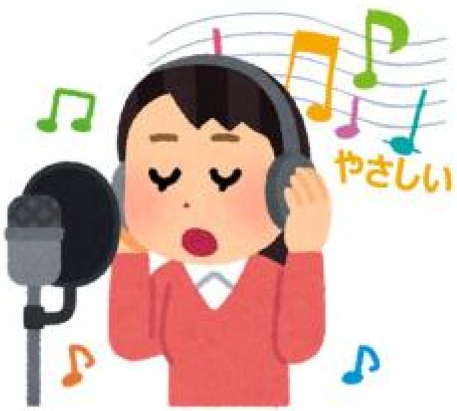


.
(　)がとてもやさしいです。

| 項目 | ( )がとてもやさしいです。 |
| --- | --- |
| 【Use all items below】<br>声<br>歌う<br>彼女<br>歌<br>【Use the items below only if necessary】<br>の<br>を<br>が | |

. Are you confident in your answer?

| 1.Not confident at all | 2.Not very confident | 3.Somewhat confident | 4.Very confident |
| --- | --- | --- | --- |
| ◯ | ◯ | ◯ | ◯ |

.

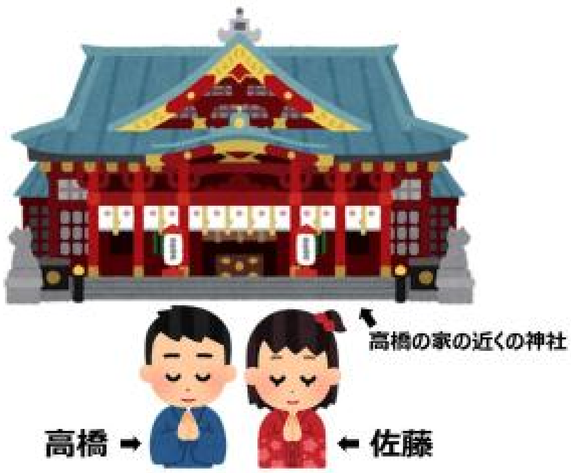


**[Mary's sentence] 高橋さんは自分の家の近くの神社に行きました。佐藤さんも行きました。**

◯ **Correct**

◯ **Incorrect**
[Optional] If possible, please enter the reason why you think it is incorrect.

◯ **Don't know**

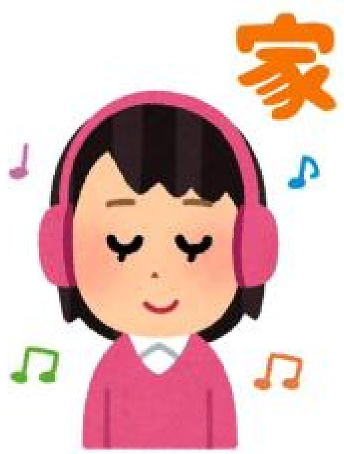


.
妹は(　　)。

| 項目 | 妹は( )。 |
|---|---|
| 【Use all items below】 | |
| 聞いています | |
| 家 | |
| 音楽 | |
| 【Use the items below only if necessary】 | |
| で | |
| を | |
| に | |

. Are you confident in your answer?

| 1.Not confident at all | 2.Not very confident | 3.Somewhat confident | 4.Very confident |
|---|---|---|---|
| ◯ | ◯ | ◯ | ◯ |

.

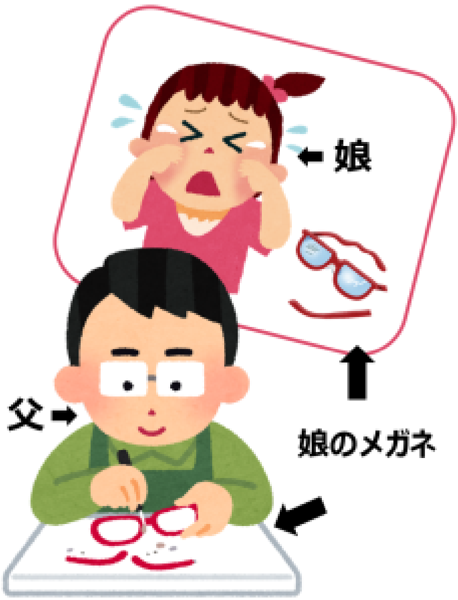


[Mary's sentence] 娘は自分のメガネを壊してしまったが、お父さんが直しました。

- ◯ **Correct**
- ◯ **Incorrect**
  [Optional] If possible, please enter the reason why you think it is incorrect.
- ◯ **Don't know**

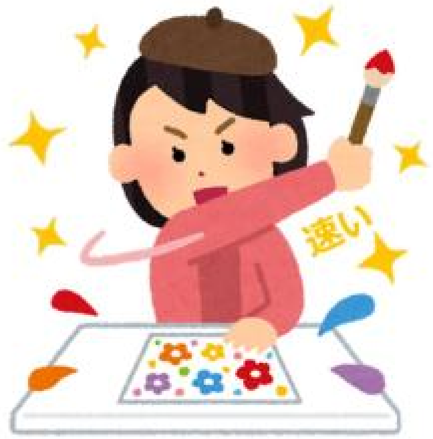


.
(　)がとても早いです。

**項目**

【Use all items below】

娘

スピード

描く

絵

【Use the items below only if necessary】

は

の

を

**( )がとても早いです。**

. Are you confident in your answer?

1.Not confident at all ◯
2.Not very confident ◯
3.Somewhat confident ◯
4.Very confident ◯

.

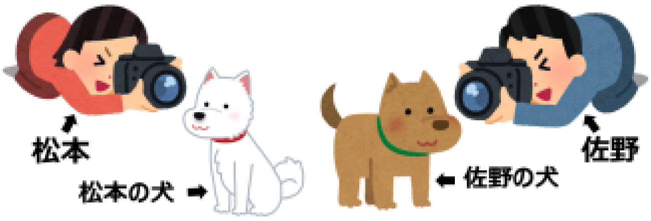


**[Mary's sentence] 】松本さんは自分の犬の写真を撮りました。佐野さんも撮りました。**

◯ **Correct**

◯ **Incorrect**

[Optional] If possible, please enter the reason why you think it is incorrect.

◯ **Don't know**

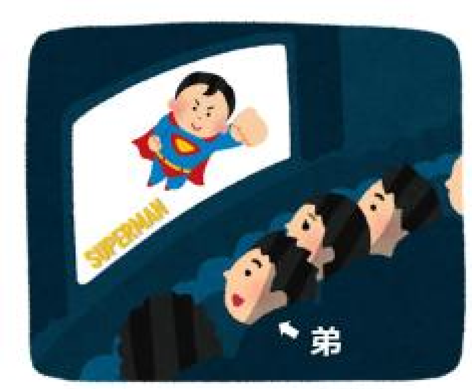


.

弟が( )。

**項目**

【Use all items below】

見ています

映画館

映画

【Use the items below only if necessary】

を

で

へ

**弟が( )。**

. Are you confident in your answer?

1.Not confident at all
2.Not very confident
3.Somewhat confident
4.Very confident

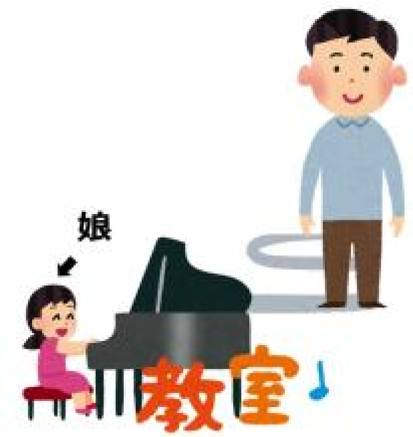


.
彼は(　)へ行きました。

**項目**

【Use all items below】

教室

娘さん

習っている

ピアノ

【Use the items below only if necessary】

の

が

を

**彼は( )へ行きました。**

. Are you confident in your answer?

| 1.Not confident at all | 2.Not very confident | 3.Somewhat confident | 4.Very confident |
|---|---|---|---|
| ○ | ○ | ○ | ○ |

.
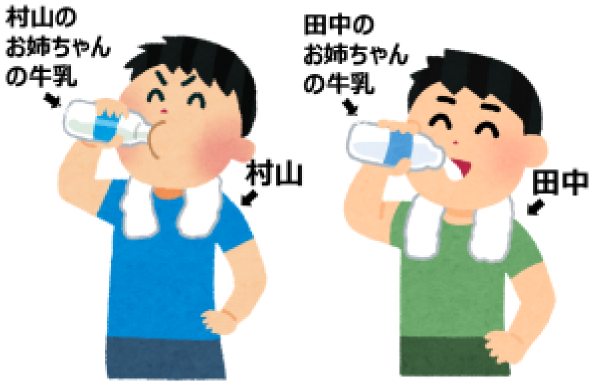


**[Mary's sentence] 村山くんは自分のお姉ちゃんの牛乳を飲みました。田中くんも飲みました。**

○ **Correct**

◯ **Incorrect**

[Optional] If possible, please enter the reason why you think it is incorrect.

◯ **Don't know**

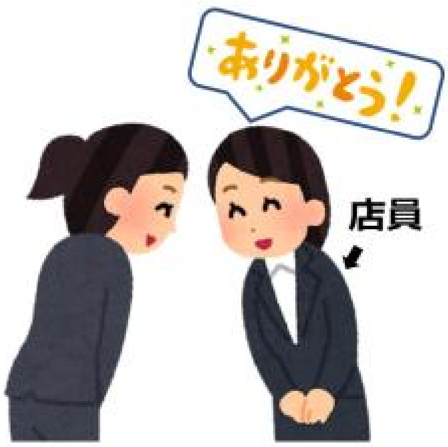


.

店員さんは(　)。

| 項目 | 店員さんは( )。 |
|---|---|
| 【Use all items below】<br>「ありがとう」<br>言いました<br>私<br>【Use the items below only if necessary】<br>に<br>と<br>を | |

. Are you confident in your answer?

| 1.Not confident at all | 2.Not very confident | 3.Somewhat confident | 4.Very confident |
|---|---|---|---|
| ◯ | ◯ | ◯ | ◯ |

.

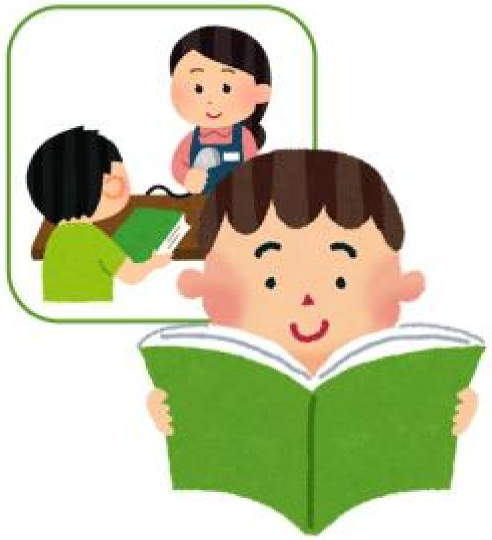

**[Mary's sentence] 男の子は本を買いましたが、まだ読み終わっていません。**

- ◯ **Correct**
- ◯ **Incorrect**
  [Optional] If possible, please enter the reason why you think it is incorrect.
- ◯ **Don't know**

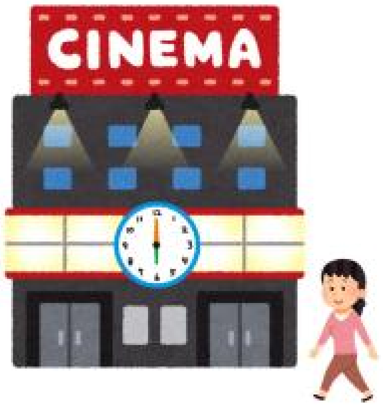


.
女の人は(　)。

| 項目 | 女の人は( )。 |
| --- | --- |
| 【Use all items below】<br>映画館<br>午後6時<br>到着しました<br>【Use the items below only if necessary】<br>に<br>に<br>で<br>へ | |

. Are you confident in your answer?

1.Not confident at all　2.Not very confident　3.Somewhat confident　4.Very confident

○ ○ ○ ○

. **English language test**

・There are 9 questions in total. There is no time limit for each question.

・Please answer all questions by yourself. We will not test your individual ability or rank you higher or lower. The purpose is to see the overall trend of correctness or incorrectness. Please do not be so concerned about the correct answers that you consult others, use dictionaries or search engines, etc., as this will distort the data.

・After you have completed the answer, press the "→" button to display the next question. Once you press "→", you cannot go back to the previous question.

**[Type: Judgment]**
Please judge whether the sentences are correct or not.
If you think it is wrong, please write a English sentence that you think is correct if possible or give us your reason in your native language (this is optional).

. **1. Mary went to school by bus.**

○ Correct

○ Incorrect
[Optional] If possible, please enter the reason why you think it is incorrect (in your native laguage).

○ Don't know

. **2. Jim realized that David ate up the last bit of cake.**

○ Correct

○ Incorrect
[Optional] If possible, please enter the reason why you think it is incorrect (in your native laguage).

○ Don't know

. **3. Mary wiped her car, and John wiped it, as well.**
***Q: What did John wipe?***

○ John's car

○ Mary's Car

○ Any of the above

○ None of the above

○ Don't know

. **4. John starts work at 9:00 a.m.**

○ Correct

- ◯ Incorrect
  [Optional] If possible, please enter the reason why you think it is incorrect (in your native laguage).
- ◯ Don't know

## . 5. Mary believes that her paper will be accepted, and John also believes that it will be accepted.
## *Q: Whose paper does John think will be accepted?*

- ◯ John's paper
- ◯ Mary's paper
- ◯ Any of the above
- ◯ None of the above
- ◯ Don't know

## . 6. They claim that Sue knew the woman.

- ◯ Correct
- ◯ Incorrect
  [Optional] If possible, please enter the reason why you think it is incorrect (in your native laguage).
- ◯ Don't know

## . 7. John saw the picture of his mother. Mary saw it, too.
## *Q: What did Marie see?*

- ◯ Photo of John's mother
- ◯ Photo of Mary's mother
- ◯ Any of the above
- ◯ None of the above
- ◯ Don't know

## . 8. Mr. White wrote a letter to his daughter.

- ◯ Correct
- ◯ Incorrect
  [Optional] If possible, please enter the reason why you think it is incorrect (in your native laguage).
- ◯ Don't know

## . 9. Kate assumed that Robert disliked the book.

- Correct
- Incorrect

  [Optional] If possible, please enter the reason why you think it is incorrect (in your native laguage).
- Don't know
